# Optimized Polyurethane/CNTs Composite for Stress-Free Two-Way Shape Memory via Training Enhancement

**DOI:** 10.3390/polym18131582

**Published:** 2026-06-25

**Authors:** Yutong Guo, Kangkang Shi, Yujie Chen, Qunfu Fan, Dongsheng Li, Hezhou Liu

**Affiliations:** 1 State Key Laboratory of Metal Matrix Composites, School of Materials Science and Engineering, Shanghai Jiao Tong University, Shanghai 200240, China; xuanyin10040@sjtu.edu.cn (Y.G.); hzliu@sjtu.edu.cn (H.L.); 2 National Key Laboratory of Ship Vibration and Noise, China Ship Scientific Research Center, Wuxi 214082, China; 3 National Engineering Research Centre of Special Equipment and Power System for Ship and Marine Engineering, Shanghai 200030, China

**Keywords:** two-way shape memory polymer, polyurethane, programmable

## Abstract

Thermally responsive shape memory polymer materials are the most widely used type of intelligent materials and have found applications in numerous fields. However, their practical utility is often limited by poor heat conduction. Carbon nanotubes (CNTs), renowned for their exceptional thermo-conductive and photothermal properties, provide a promising solution. In this study, CNTs were integrated into polyurethane prepared by stepwise polymerization method, using hydroxyl terminated polycaprolactone (PCL-diOH), poly(ethylene glycol) (PEG) and hexamethylene diisocyanate (HDI). The resulting polyurethane composite material exhibits remarkable mechanical strength, enhanced thermal conductivity, and superior shape memory performance. Notably, it demonstrates a form of training enhancement phenomenon, which shows higher mechanical properties. And the composite could achieve stress-free two-way shape memory behavior after cyclic stretching process. Additionally, this composite material can exhibit “vitrimer” material properties at higher temperatures (110 °C), allowing for shape reprogramming. The carbon nanotube-reinforced composite material can achieve remote and precise manipulation under light stimulation. By combining the composite material with a metal thermally conductive layer, a multi-layer structure with shape memory properties can be prepared, which can achieve two-way shape memory behavior under electrical and light stimulation, further expanding the application potential of the composite material in the real world.

## 1. Introduction

Shape memory polymers (SMPs) are a fascinating class of smart materials that can change their shape in a controlled manner when exposed to specific external stimulations like heat [1,2,3,4], light [4,5,6], magnetic fields [7], or electric fields [8,9,10]. Shape memory polymers stand out due to advantages like light weight, easy processing, low cost, deformability, etc. [11,12]. These properties make them highly promising for applications in flexible robotics [13,14,15,16], soft actuators [17,18,19,20,21], biomedicine [22,23,24], self-healing materials [25], and aerospace [26]. Shape memory polyurethane (SMPU), as a typical heat-responsive shape memory polymer, has become one of the research hotspots in recent years [27]. Kim et al. prepared a shape memory polyurethane using catechol and poly(ethylene glycol), which achieved improved mechanical properties, programmable crystallization, and solar-driven shape recovery through the coordination between catechol and Fe^3+^ ions [28]. Chu et al. developed an injectable polyurethane–gelatin material system that exhibits shape memory behavior under both solution and temperature stimulation. Furthermore, by controlling the crosslinking density of the polymer system, a bilayer-structured actuator capable of two-way shape memory behavior was fabricated [29]. Yang et al. modified thermoplastic polyurethane with carbon black to enhance the electrical conductivity and mechanical properties of the composite system, enabling shape memory behavior under both thermal and electrical stimulation [30]. Among various driving methods, thermal driving is the easiest form to implement. Therefore, thermally responsive shape memory polymers are the most extensively researched. They can swiftly react to temperature changes, show benefits like rapid response, high energy efficiency, and straightforward activation. However, there were some limitations for thermal response shape memory materials, such as the need for an external heat source and the inability to achieve precise or remote control. In addition, most shape memory polymer materials have low thermal conductivity, which can easily lead to energy waste. Additionally, the low thermal diffusivity leads to prolonged response and recovery times for the materials.

In practical applications, thermally responsive shape memory polymers can be heated using alternative methods like electricity or light, allowing for remote control [31,32,33,34]. However, the practical application of these polymers is hindered by their low thermal conductivity and inefficient photothermal or electrothermal conversion. By doping photothermal particles or conductive materials into polymers, their thermal conductivity and photothermal conversion performance can be improved [35,36]. For example, coating the polymer surface with black or photothermal materials can enhance its photothermal performance, thereby increasing the polymer’s temperature under irradiation conditions [37]. However, the doped particles may adversely affect the basic properties of the polymer, such as mechanical properties and shape memory properties. Previous studies have investigated that carbon nanotubes, MXene, and carbon black (CB) and other reinforcing phases are used to improve the properties of shape memory composite materials [38]. For example, Tekay et al. composited multi-walled carbon nanotubes into a copolyester thermoplastic elastomer (COPE) to prepare a shape memory polyurethane composite material that responds to electrical and light stimuli [39]; Rahmatabadi et al. prepared a carbon nanotube-reinforced polyurethane 4D printing material, which exhibited excellent mechanical and shape memory properties [40]; Zhang et al. prepared an electrically responsive shape memory composite material by simultaneously adding carbon black and carbon nanotubes to an epoxy resin matrix [37]. However, these works share some common issues, such as the high hardness of the prepared composite materials, which can only undergo bending shape memory deformation and cannot achieve shape memory behavior after stretching. Moreover, the weak actuation force, along with a slow response and poor energy density, forces the use of higher voltages and light intensities to achieve adequate performance. How to prepare a polymer composite material that can achieve remote precise control while possessing excellent mechanical properties and shape memory properties has prominent research significance. Furthermore, the introduction of fillers can affect the segmental mobility of shape memory polymers to some extent, thereby influencing their shape memory performance. However, most shape memory composites are limited to unidirectional shape memory behavior, which falls far short of practical requirements. Therefore, it is challenging to prepare high-performance two-way shape memory composite materials through molecular design and material component design.

Carbon-based materials exhibit significant light absorption capabilities and can convert absorbed light into thermal energy through lattice vibrations. These materials have loose electrons in their π orbitals, which can be excited under near-infrared light irradiation. During the relaxation process of excited electrons back to the ground state, the absorbed energy will be released in the form of thermal energy [39]. Carbon nanotubes, as one-dimensional carbon-based nanomaterials, have an excellent aspect ratio, specific strength, specific toughness, conductivity, and photothermal properties. As a reinforcing phase, they can significantly enhance the mechanical and photothermal properties of the polymer composites. However, due to strong Van der Waals interactions, carbon nanotubes are prone to aggregation and difficult to disperse in polymer matrices, which can have a negative impact on polymer properties. Surface modification of carbon nanotubes can effectively improve their dispersibility and enhance the direct interface bonding effect between carbon nanotubes and resin matrix.

The purpose of this work is to prepare a flexible driving material with excellent mechanical properties, fast response, remote control, and reversible motion, and explore its potential applications in the fields of flexible actuators and artificial muscles. Based on the excellent performance of carbon nanotubes, in this study, the surface-modified carbon nanotubes were used as reinforcing phases to improve the thermal conductivity and photothermal conversion efficiency of polyurethane-based composites. The modified carbon nanotubes can be more evenly dispersed in polyurethane composite materials, serving as crystal nuclei and skeletons to promote polyurethane crystallization, while to some extent limiting the activity of polymer segments and enhancing the mechanical properties of polyurethane. In addition, carbon nanotubes gradually exhibit an orientation trend along the stretching direction during the stretching process of composite materials, resulting in a training enhancement effect during repeated shape memory cycles. Due to the presence of carbon nanotubes and the limitation of the activity of some polymer segments, the stability of this polyurethane composite material increases at high temperatures, exhibiting a behavior similar to that of a “vitrimer” material, blending the properties of thermosetting and thermoplastic polyurethane polymers. In this composite system, the reinforcing phase and the polymer matrix exhibit a synergistic effect, which not only reinforces the material but also enables shape memory re-programmability and recyclability. After multiple shape memory cycles, the composite exhibits stress-free two-way shape memory behavior, significantly enhancing its practical applicability. Furthermore, we have prepared a double-layer structure actuator that can achieve reversible shape changes by controlling the voltage or light intensity, expanding the practical application potential of composite materials. The shape memory polyurethane boasts outstanding mechanical strength and energy density, making it ideal for real applications.

## 2. Materials and Methods

### 2.1. Materials

Hydroxyl terminated PCL-diOH (Mn = 20,000) was obtained from the Shanghai D & B laboratory (Shanghai, China). PEG (Mn = 2000) was purchased from Sinopharm Chemical Reagent (Shanghai, China). Hexamethylene diisocyanate (HDI) was purchased from Shanghai Macklin Biochemical Co (Shanghai, China). The multi-walled carbon nanotubes were purchased from Leyan Company (Shanghai, China). Dipropylene glycol dimethyl ether (DPGDME) was purchased from Shanghai Titan Technology Co., Ltd (Shanghai, China). 4A molecular sieve was purchased from Energy Chemical Co., Ltd (Shanghai, China). N,N,N′,N′-tetrakis(2-hydroxypropyl)ethylenediamine (HPED) and Triethanolamine (TEA) were purchased from Shanghai Aladdin Biochemical Technology Co., Ltd. (Shanghai, China). The concentrated sulfuric acid (the concentration is 98%), concentrated nitric acid (the concentration is 68%), carbon black (CB_S_, Super P) and Fe_3_O_4_ nano partials, (the diameter is 100 nm) were sourced from Shanghai Titan Technology Co., Ltd. (Shanghai, China). Reagents were used as received except under specific instructions. All of the reagents need to be stored in a dry condition.

### 2.2. Preparation of the m-CNTs

The multi-walled carbon nanotubes need to be modified by acidification. Prepare a mixed solution of concentrated sulfuric acid and concentrated nitric acid in the ratio of 3:1. Weigh 1 g of CNTs, acidify at room temperature for 24 h, and then stand overnight. Remove the upper acid solution. Then wash the CNTs with deionized water, and let it stand for layering again, remove the upper solution. Repeat the washing process until the pH value of the solution is neutral. The modified carbon nanotubes m-CNTs were obtained by suction filtration and drying at 80 °C.

### 2.3. Preparation of the Shape Memory Polyurethane Composite

The polyurethane was fabricated by a stepwise polymerization method. First, polycaprolactone and HDI were mixed in DPGDME with a molar ratio of 1:2 and heated at 80 °C for 10 min. Next, poly(ethylene glycol), HPED, and TEA were added, and the mixture was allowed to react for another 10 min. After that, m-CNTs were introduced and evenly dispersed. The mixture was then degassed under vacuum, poured into a mold, and heated at 80 °C to remove the solvent, resulting in the formation of shape memory polyurethane. Note that the molar ratio of polycaprolactone to poly(ethylene glycol) is 7:3, and all reagents need to undergo dehydration treatment before use. It should be noted that the stepwise polymerization method may generate waste, and the introduction of nanoparticles may result in toxicity and leakage. Therefore, the entire preparation process should be carried out in equipment with exhaust gas treatment. The waste liquid generated during the preparation process should be strictly recycled and treated in accordance with relevant environmental standards.

### 2.4. Preparation of the Muti-Layer Structure Materials

First, the shape memory polyurethane is cut into the desired form. It is then stretched at a temperature of 70 °C. After stretching, the temperature is reduced while keeping the stress constant, which locks the material into its new shape. Finally, this programmed SMPU is bonded to a metal layer, creating a composite material with a two-layer structure.

### 2.5. Characterizations

The scanning electron microscope (SEM) experiment was carried out on a Carl Zeiss Supra 55 scanning electron microscopes (Carl Zeiss AG, Oberkochen, Germany). A 10 nm thick gold was sputtered onto the structure prior to the SEM observation. The FTIR spectrum was measured by Spectrum 100 FTIR Spectrometer (PerkinElmer Inc., Waltham, MA, USA), and the scan range was 800–4000 cm^−1^. The crystallization was characterized by X-ray diffractometer (XRD, Cu target at room temperature, Bruker Corporation, Billerica, MA, USA), and the testing range was 5–90°, scanning speed was 5°/min. The Raman spectrum of the sample was detected using a Raman spectrometer (DXR, Thermo Fisher Scientific, Waltham, MA, USA) with a scanning range of 100–3000 cm^−1^ and a laser wavelength of 780 nm. The thermal properties were characterized by thermogravimetric analyzer (TGA, TGA550, American TA Instrument, New Castle, DE, USA) and differential scanning calorimeter (DSC, DSC250, American TA Instrument, New Castle, DE, USA), and the temperature rising/falling rate was 10 °C/min, nitrogen atmosphere. Mechanical properties were analyzed by microcomputer controlled electronic universal testing machine (LD23, Shenzhen, China) at room temperature. The stretch samples were dumbbell-shaped which with size for parallel part 10 mm (length) × 4 mm (width) × 0.25 mm (thickness). The tensile rate was 10 mm/min. The dynamic mechanical analysis and shape memory performance were tested by dynamic mechanical thermal analyzer (DMA 850, American TA Instrument, New Castle, DE, USA). The thermal diffusion coefficient, thermal conductivity, and heat capacity of the test material were measured using a laser thermal conductivity meter (LFA 467, NETZSCH, Selb, Germany). The sample needs to be cut into circular thin sheets and subjected to carbon spraying treatment. Other conditions were described in the analysis section of the result and Supporting Information.

## 3. Results and Discussion

### 3.1. The Properties of Polyurethane Composite

**Figure 1:**
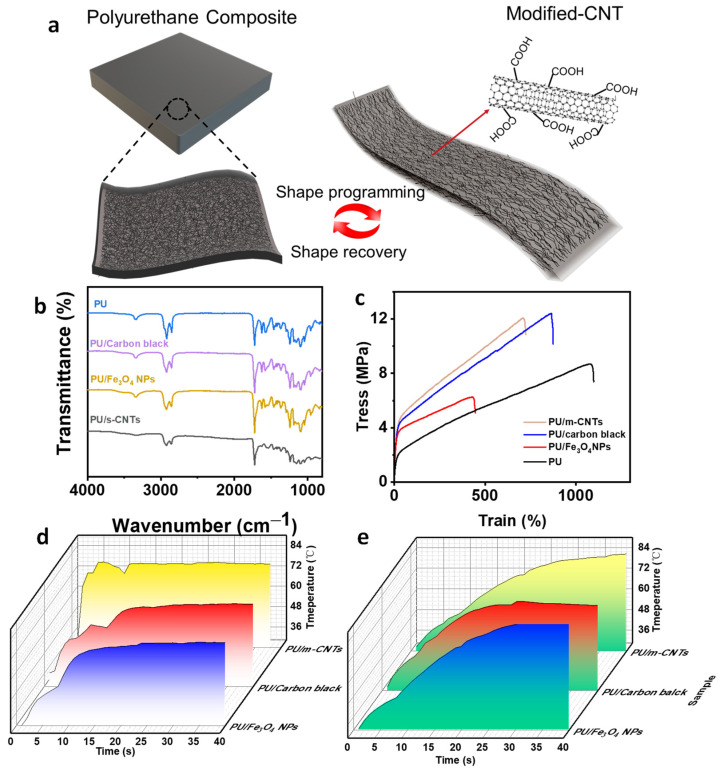
(**a**) Schematic illustration of polyurethane; (**b**) the Fourier transform infrared spectroscopy (FTIR) data of the polyurethane with different fillers; (**c**) mechanical properties of polyurethane with different fillers; the surface temperature change of different composites in (**d**) 80 °C condition and (**e**) light irradiation.

In this work, the shape memory polyurethane composite is mainly composed of polycaprolactone and poly(ethylene glycol), doped with modified carbon nanotubes (m-CNTs), as shown in Figure 1a. Polycaprolactone is terminated by hydroxyl groups. The hydroxyl groups of polycaprolactone and poly(ethylene glycol) can react with isocyanate groups to form urethane bonds, as shown in Section S1 (Supporting Information). This shape memory polyurethane is a thermally responsive material, which can achieve shape programming and recovery under different temperature conditions. In our previous work, we conducted preliminary research on the shape memory performance of this material system [41]. To meet the needs of remote and precise control in practical applications, photothermal particles are introduced as fillers to modify the thermal conductivity and photothermal properties of the polyurethane, enabling SMPU to respond to light and electrical stimuli. In this work, the effects of carbon black, Fe_3_O_4_ nanoparticles, and m-CNTs on the properties of polyurethane were studied, and the material morphology is shown in Figure S1, where the content of the three particles is 5 wt%. Figure 1b shows the FTIR spectra of different polyurethane composites, which shows that the peak at 1143 cm^−1^ is the vibrational absorption of the ether C-O-C bond, and the peak at 1725 cm^−1^ is the vibrational absorption of the ester bond. And the strong peaks of -OH in PCL-diOH and PEG disappear. And the peaks at 1580 cm^−1^ show the bending vibration and the secondary amide II band vibration absorption of NH bond, proving the formation of the urethane bond. It is worth noting that there are carboxyl groups on the surface of the modified carbon nanotubes, which may react with the hydroxyl groups in the polymer to form ester bonds, thereby consuming the abundant hydroxyl groups and causing a decrease in peak intensity at 3340 cm^−1^. In addition, the introduction of photothermal particles did not affect the molecular structure of polyurethane.

Furthermore, it should be noted that carbon nanotubes (CNTs) are difficult to disperse in polyurethane due to severe entanglement and fewer surface functional groups. Therefore, it is necessary to use a mixture of concentrated acids to modify the surface of CNTs to enhance their dispersibility. Figure S2 shows the FTIR spectra of CNTs before and after modification. And it could be found that the modified CNTs show higher peak intensity at 3439 cm^−1^. In addition, the peak at 1650 cm^−1^ corresponds to the stretching vibration of the C=O bond, and the characteristic peak at 1128 cm^−1^ corresponds to the C-O-C bond. The increase in peak intensity at these positions proves the increase in carboxyl content in carbon nanotubes. Additionally, the peak at 1384 cm^−1^ corresponds to the symmetrical bending vibration of methyl. Figure S3 shows the SEM pictures of CNTs before and after modification, where the m-CNTs show better uniformity compared to aggregated CNTs. And after acid treatment, m-CNTs can achieve relatively uniform dispersion in polyurethane, as shown in Figure S4g–i. In addition, Figure S4a–c show the surface and interface images of PU/CBs samples, indicating that carbon black particles were observed to form aggregates within the polyurethane matrix. Nevertheless, these aggregates did not accumulate in any particular region but were evenly distributed throughout the entire matrix. In contrast, due to its high density, Fe_3_O_4_ nanoparticles are prone to settling during the polyurethane polymerization process, resulting in their deposition on one side of the polyurethane matrix, as shown in Figure S4d–f. Figure S5 shows the influence of different nano-photothermal particles on the thermogravimetric properties of the polyurethane composites, revealing no significant differences among the samples. The introduction of enhanced particles will slightly increase the initial decomposition temperature of the composite material, with carbon nanotubes showing the greatest increase. The addition of carbon nanotubes and carbon black will reduce the fracture elongation and increase the tensile strength of polyurethane composite materials. However, Fe_3_O_4_ nanoparticles have a significant weakening effect on the mechanical properties of the composites, with both tensile strength and elongation at break showing a considerable decrease compared to composites doped with carbon black and m-CNTs, as shown in Figure 1c.

In addition, Figure 1d,e, Figures S6 and S7 show the surface temperature changes over time of different polyurethane composite materials in an environment of 80 °C and under the condition of 0.8 W/cm^2^ light irradiation, respectively. From the figures, the m-CNTs-doped polyurethane composite has the highest thermal conductivity rate and photothermal conversion efficiency in high-temperature environments. To verify this point, this work characterized the thermal conductivity of different polyurethane composite, and the results are shown in Table S1. From the characterization results above, it can be seen that the modified carbon nanotubes can be relatively uniformly dispersed in the polyurethane composite, improving the mechanical properties of the composite material. Compared to carbon black and Fe_3_O_4_ nanoparticles, carbon nanotubes can effectively increase the thermal diffusivity of polyurethane composites, thereby enabling shape memory composites to achieve faster response speeds. Under moderate intensity light, carbon nanotube modified polyurethane composite materials could also achieve rapid heating and remote control. After comprehensive consideration, the m-CNTs-doped polyurethane composite has the maximum thermal conductivity efficiency, which is consistent with the results in Figure 1.

**Figure 2:**
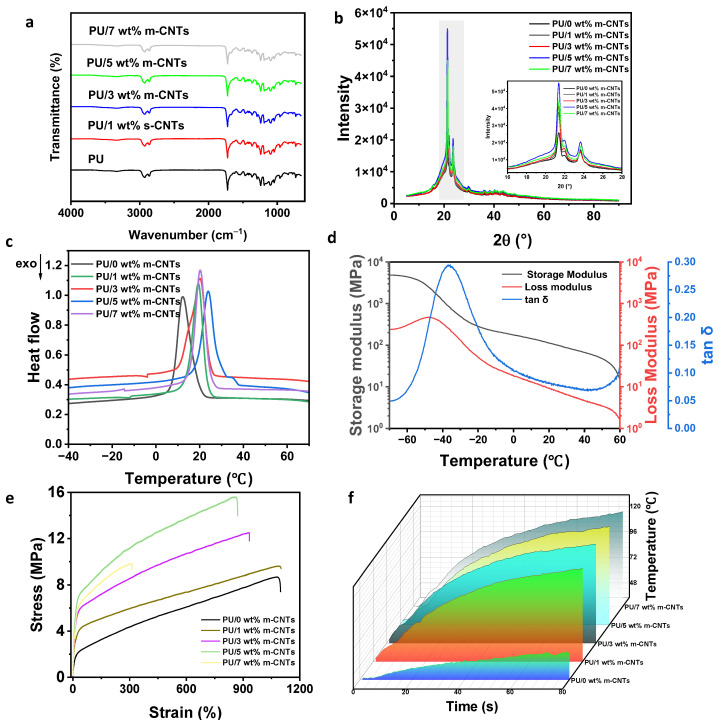
(**a**) The FTIR data, (**b**) X-ray diffraction (XRD) data, (**c**) differential scanning calorimetry (DSC) data, and (**e**) mechanical properties of polyurethane composite with different m-CNTs contents. (**d**) The dynamic mechanical thermal analysis (DMA) data of PU/5 wt% m-CNTs; (**f**) the surface temperature change of polyurethane composite with different m-CNTs contents.

Next, this work further investigates the influence of different carbon nanotube contents on the properties of shape memory polyurethane composite. Figure 2a shows the FITR curves of polyurethane composite with different m-CNTs contents, which could be analyzed that the m-CNTs take a little influence with the polymer molecular structure. It is worth noting that after surface modification, there will be some carboxyl groups on the surface of CNTs, some of which may form hydrogen bonds with polyurethane polymer chains, increasing the overall hydrogen bond density of the composite material system. However, due to the steric hindrance effect generated by m-CNTs, the ordered arrangement of some polymer segments is disrupted, resulting in a decrease in the density of ordered hydrogen bonds, an increase in the density of disordered hydrogen bonds, and a decrease in overall hydrogen bond strength. This can be supported by the FTIR spectral data of composite materials. Figure S8 shows the magnified curve of Figure 2a at 3330 cm^−1^. Through observation, it can be found that with the increase in m-CNTs content, the peak width and peak strength of the composite material at 3330 cm^−1^ increase, corresponding to an increase in hydrogen bond density, but a decrease in ordered hydrogen bond density. The crystal structure of the polymer has been characterized by XRD and is shown in Figure 2b. The peaks at 21.37°, 21.95°, and 23.66° are identified as the crystalline peaks of polycaprolactone, specifically corresponding to the (110), (111), and (200) crystal planes, respectively. A closer look reveals that the intensity of these crystalline peaks in the polyurethane composite increases with the addition of carbon nanotubes (m-CNTs), peaking at a 5 wt% m-CNTs content. Beyond this point, further increases in m-CNTs content lead to a reduction in peak intensity. This phenomenon suggests that a small amount of m-CNTs can enhance polymer crystallization. m-CNTs, with their high specific surface area, act as effective nucleating agents, providing sites for polymer crystallization. They also help in organizing polymer chains on their surfaces, speeding up the crystallization process. Moreover, m-CNTs improve the thermal conductivity of the composite, facilitating faster cooling and thus promoting crystallization. However, excessive m-CNTs content can cause agglomeration within the polyurethane, disrupting crystallization and reducing peak intensity in XRD. This observation is supported by DSC analysis, as illustrated in Figure 2c and Figure S9. Figure 2c demonstrates that the crystallization temperature of the composite rises with increasing m-CNTs content, reaching a maximum of about 23.6 °C for PU/5 wt% m-CNTs. Further increases in m-CNTs content, however, result in a drop in crystallization temperature, aligning with the XRD findings. Additionally, the dynamic mechanical properties of the 5 wt% m-CNTs polyurethane composites were examined, as shown in Figure 2d. Compared to the m-CNTs-free polyurethane (Figure S10), the composite exhibits higher storage modulus and loss factor, and its glass transition temperature increases from −41 °C to −23 °C.

In this work, we characterized Raman spectroscopy on unmodified carbon nanotubes, modified carbon nanotubes, and polyurethane composites containing modified carbon nanotubes, as shown in Figure S11. From the figure, it can be seen that CNTs, m-CNTs, and PU/m-CNTs all exhibit typical graphite carbon atom characteristic peaks, where 1350 cm^−1^ represents the D band and 1580 cm^−1^ represents the SP^2^ hybridized G band. By comparing the intensities of the D and G peaks, a rough assessment of the degree of surface defects in CNTs can be made. The I_D_/I_G_ of CNTs is 0.94, indicating that the carbon nanotube itself has many defects. After modification, the I_D_/I_G_ value of m-CNTs increased to 0.96, indicating that the surface defects of the modified carbon nanotubes further increased. The I_D_/I_G_ value of PU/m-CNTs is 1.03, indicating that m-CNTs have achieved relatively good dispersion in the polyurethane matrix, and the combination of polyurethane molecular segments with m-CNTs has disrupted the regularity of carbon nanotubes and increased their surface defects. The mechanical properties and thermal conductivity of polyurethane composite with different m-CNTs contents were also studied. Figure 2e shows the stress–strain curves of different polyurethane composite. It can be seen from the figures that as the m-CNTs content increases, the tensile strength of the composite increases, while the elongation at break decreases. This is because a small amount of m-CNTs can promote polymer crystallization, and it will orient along the stretching direction during the stretching process, thereby enhancing the tensile strength of the material. The composite with 5 wt% m-CNTs contents shows best mechanical properties with the 857% elongation at break and 15.6 MPa tensile strength. Figure S12 shows the pictures of PU/5 wt% m-CNTs before and after stretching. When the m-CNTs content is too high (7 wt%), the overall mechanical properties are significantly affected, and the elongation at break and tensile strength are greatly reduced. These excessive m-CNTs may agglomerate in the polyurethane and affect the polymerization process of the material. Figure 2f shows the photothermal conversion efficiency of different polyurethane composite. Under the same intensity of light, the surface temperature changes of the materials are shown. It can be seen that the introduction of m-CNTs can effectively improve the photothermal conversion efficiency of the composite. When the m-CNTs content is ≥3 wt%, the heating rate and the final maximum temperature reached by polyurethane composite with different m-CNTs contents are similar. In summary, 5 wt% m-CNTs was selected as the optimal photothermal particle doping ratio in this work for subsequent research.

### 3.2. The Shape Memory Properties of Polyurethane Composite

**Figure 3:**
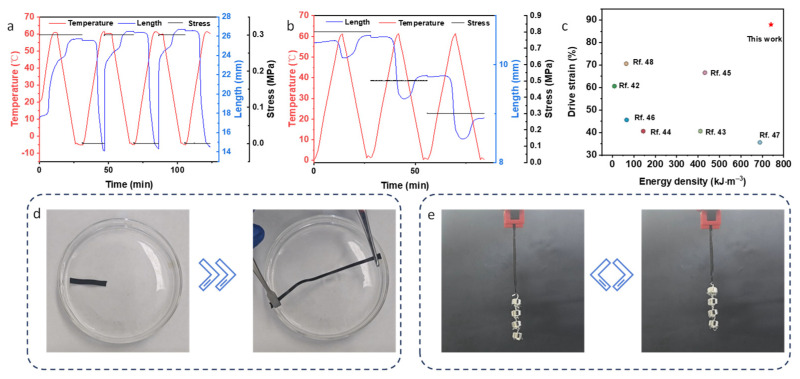
(**a**) The shape memory process DMA data of PU/5 wt% m-CNTs; (**b**) the DMA data shows the Quasi two-way shape memory process of polyurethane composite that under load; (**c**) the comparison of composite material properties with other literature; (**d**) shows the shape memory process under different temperature conditions and (**e**) shows the quasi-two-way shape memory process.

We explored the shape memory capabilities of a shape memory polyurethane composite, illustrated in Figure 3a. When the material is heated beyond its critical temperature (melting temperature T_m_), its molecular chains become more mobile, enabling the polyurethane to stretch along the direction of applied stress, leading to deformation. By keeping the external stress constant and cooling the material, the shape can be fixed—a process called shape programming. During this phase, the external stress and energy are stored within the polyurethane as entropy increases. Later, when the external stress is removed and the material is heated above the critical temperature again, the polymer chains unfreeze, and the stored stress and energy are released under entropy-driven conditions, causing the material to revert to its original stable state—a process known as shape recovery. This cycle could be repeated multiple times, as demonstrated in Figure 3d, and Video S1.

Notably, in Figure 3a, when the polyurethane composite is cooled under external stress, a secondary elongation is observed. This phenomenon arises because the applied stress promotes oriented crystallization of the polyurethane along the stress direction, leading to an increase in length—a phenomenon termed crystallization-induced elongation (CIE). Utilizing this property, when a constant external stress is applied to the polyurethan composite, it can exhibit a reversible shape memory process under varying temperature conditions, as depicted in Figure 3b. After shape programming, if the load is maintained, raising the temperature to the critical point causes the composite to contract toward its original length. Conversely, lowering the temperature causes the material to crystallize along the stress direction, increasing its length. This quasi-two-way shape memory behavior can be cycled by altering temperature conditions, as shown in Figure 3e and Video S2.

In addition, this work also tested the shape fixation rate (R_f_), shape recovery rate (R_r_), energy density (W), and power density (P) of the polyurethane composite. The formulas used for these calculations are detailed in the Supporting Information, and the results are summarized in Tables S2 and S3. The data reveals that adding carbon nanotubes (m-CNTs) slightly reduces both the shape fixation and recovery rates of the shape memory polyurethane, though these values still remain impressively high, at over 96% and 92%, respectively. Interestingly, incorporating an optimal amount of m-CNTs enhances both the power density and energy density of the composite. This improvement is attributed to m-CNTs’ ability to enhance the polyurethane’s crystallinity. The best performance was observed in the PU/5 wt% m-CNTs sample, which achieved energy and power densities of 679.39 kJ·m^−3^ and 468.55 W·kg^−1^, respectively. Figure 3c shows the performance of this work and some studies in terms of driving strain and power density [42,43,44,45,46,47,48].

### 3.3. The Topological Transition Performance of the Polyurethane Composite

**Figure 4:**
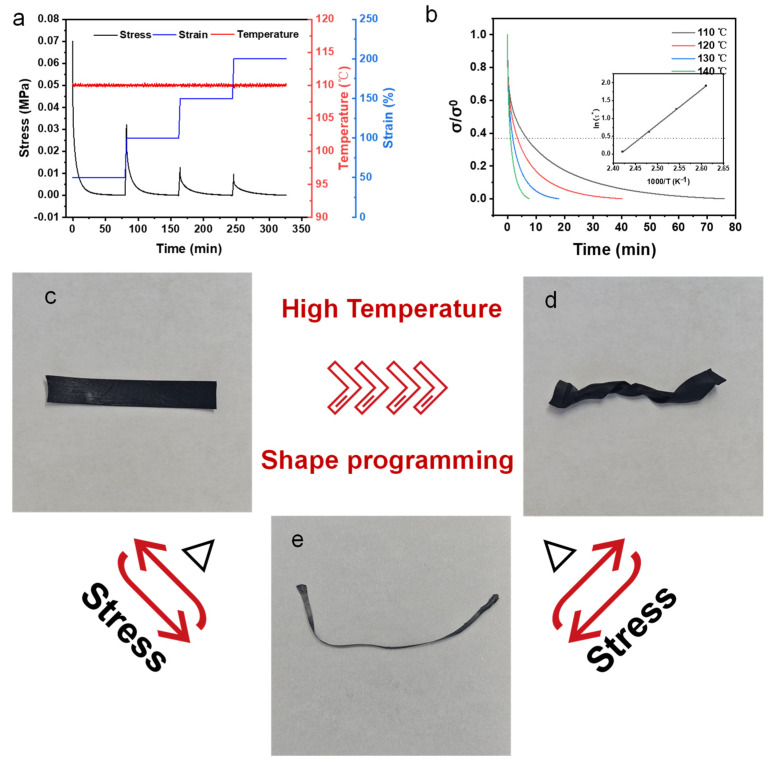
Stress relaxation of polyurethane under (**a**) different stresses and (**b**) different temperatures; (**c**–**e**) secondary shape programming process of shape memory polyurethane composites and their shape memory process.

It is worth noting that our polyurethane composite can exhibit typical “vitrimer” material characteristics, that is, it possesses both thermosetting and thermoplastic polymer properties. Under specific temperature conditions, it can undergo ester bond exchange reactions, thereby achieving rearrangement of the polymer network, which is shown in Figure S14b. This polyurethane material uses HPED and TEA as crosslinking agents during polymerization, thus having a crosslinked polymer network and exhibiting thermosetting polymer material characteristics. Stress relaxation tests were conducted at 80 °C, and the results are shown in Figure S13. After prolonged relaxation, the polyurethane composite can still maintain a certain level of internal stress, demonstrating typical thermosetting material properties. However, DMA testing of the polyurethane composite reveals that the loss factor (tan δ) undergoes significant changes as the temperature approaches the melting point. This is attributed to the increased mobility of polymer chain segments at this temperature, which causes notable changes in both the storage and loss modulus. When the temperature of the material system is further increased, it can be observed that tanδ also undergoes very noticeable changes around 110 °C. At this temperature, the polyurethane undergoes transesterification reactions, leading to rearrangement of the polymer network, as shown in Figure S14a. To verify this point, stress relaxation at 110 °C was characterized and shown in Figure 4a. From this figure, it can be seen that under this temperature condition, the stress of the polyurethane can relax to 0 MPa after a certain period of time, exhibiting thermoplastic material characteristics. s-CNT plays a crucial role in this composite material. It is uniformly dispersed in the polyurethane matrix and serves as a skeleton for a portion of the composite material, which could limit the activity of polymer segments. Therefore, the polymer maintains the stability of its molecular structure at high temperatures, thereby achieving chain rearrangement. If the presence of m-CNTs is absent, the activity of the polyurethane system at high temperatures is significantly enhanced. Under the same testing conditions, its storage modulus drops sharply at 70 °C, and the fluctuation of the DMA curve increases, as shown in Figure S15.

Vitrimer materials can undergo rearrangement of the polymer network, i.e., a topological transformation process, while maintaining the overall crosslinking density unchanged. This process satisfies the Arrhenius equation. To demonstrate that this reaction process is a topological transformation, we characterized the stress relaxation process of polyurethane composite under different temperature conditions, and the results are shown in Figure 4b. The relationship between the relaxation time τ and the reaction rate constant κ is shown in formulas (7) (Supporting Information). By plotting, it can be seen that lnκ and 1/T exhibit a linear relationship, satisfying the Arrhenius equation, and the reaction activation energy E_a_ can be calculated to be approximately 81.01 kJ·mol^−1^. Where the σ is real time internal stress and σ^0^ is initial internal stress. The dashed line in Figure 4b represents the value is equal to 1/e. τ* is the relaxation time of the material at different temperatures at the dashed line.

Based on the property of the polyurethane composite that can undergo topological transformation at high temperatures, it can achieve a secondary programming shape memory process, as shown in Figure 4c–e. When the polyurethane composite obtains a temporary shape, it can return to its original state under thermal stimulation (higher than T_m_), the process shown in Figure 4c,e. While if the polyurethane is programmed at a higher temperature, e.g., higher than 110 °C, the polymer network will rearrange and the original state will change into a new shape, as shown in Figure 4d. Therefore, when the shape memory behavior occurs again, the polyurethane composite will return to the new original shape, this process is shown in Figure 4d,e. The entire process is fully demonstrated in Video S1. In addition, based on its topological transformation properties, it can also achieve the recycling of thermosetting polyurethane materials, as shown in Figure S16. The tensile strength retention rate of the recycled samples is 71%.

### 3.4. The Training Enhancement Phenomenon of Polyurethane Composite

**Figure 5:**
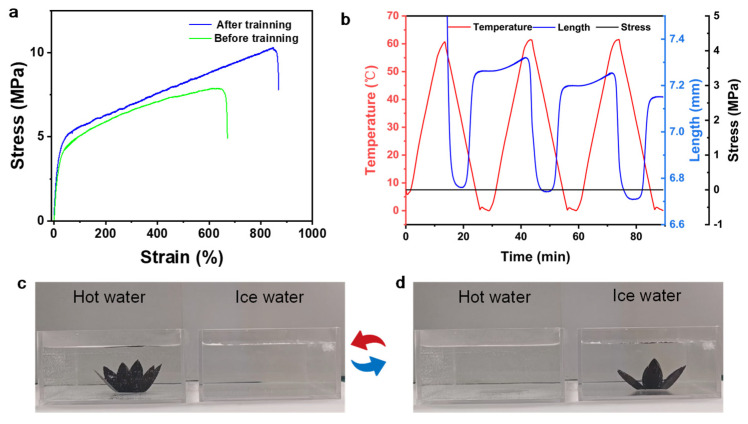
(**a**) Stress–strain curves of polyurethane composite before and after training; (**b**) DMA curves of stress-free two-way shape memory behavior; (**c**,**d**) stress-free two-way shape memory behavior.

The shape memory polyurethane composite exhibits a phenomenon of training enhancement. After undergoing multiple shape memory processes (≥8 times), the mechanical properties of the same composite show significant improvement. Figure 5a shows the stress–strain curves of the same polyurethane composite material before and after cyclic training. It clearly demonstrates that the polyurethane composite after cyclic training has higher tensile strength and elongation at break. This is attributed to the gradual alignment of m-CNTs along the stretching direction during successive shape memory cycles. Based on the excellent axial tensile properties of m-CNTs, they can effectively enhance the mechanical properties of the composite. As shown in Figure S17, the Young’s modulus of the polyurethane after cyclic stretching is significantly higher than that in the original state. In addition, Figure 3 indicates that the polyurethane composite can achieve quasi-two-way shape memory behavior under certain external stress conditions, but if the external stress is completely removed, it cannot exhibit a reversible two-way shape memory process. The reason is that the deformation amplitude based on CIE, as shown in Figure 3a, is very small, and without stress conditions, it cannot induce its directional crystallization. However, after cyclic testing, the m-CNTs in the polyurethane will align, and the m-CNTs can act as nucleating agents during the cooling process of the polymer; they can induce the polymer to crystallize along the original stretching direction without stress, thus exhibiting stress-free two-way shape memory behavior. Figure 5b shows the DMA test results of the two-way shape memory process under stress-free conditions. Figure 5c,d and Video S3 demonstrate that the trained polyurethane composite acting as a “muscle” can drive a flower to achieve a reversible stress-free two-way shape memory process under different temperature stimuli.

In addition, we also tested the cyclic stability of the composite material. Firstly, 20 complete shape memory processes were performed on different samples, and then their shape memory performance was tested. The results are shown in Table S4. It can be seen that after multiple shape memory cycles, the shape fixation rate and recovery rate of the composite material have both decreased to a certain extent. However, except for PU/7 wt% m-CNTs, the overall stability of the other samples can still be maintained at over 92%, proving that the composite material system has certain cyclic stability. Subsequently, we conducted 500 cyclic tensile tests on PU/5 wt% m-CNTs using an electronic testing machine, and the results are shown in Figure S18. As shown in the figure, the overall mechanical properties of the composite are largely retained after multiple stretching cycles, with only a 6% reduction in tensile strength. We characterized the samples after cyclic testing using DMA and DSC, and the results are shown in Figures S19 and S20, respectively. The sample can still achieve stress-free two-way shape memory behavior after multiple cycles of stretching, proving that its internal m-CNTs can still maintain a certain degree of orientation after multiple cycles. However, there was a significant decrease in the melting temperature of the sample, which was due to multiple cycles of stretching causing the crystallinity of some internal chain segments of the sample to be disrupted, resulting in a decrease in the overall crystallinity of the material and a decrease in its melting temperature. In summary, the composite material system exhibits relatively good cyclic stability.

### 3.5. The Multiple Driving Modes of Multi-Layer Structure Materials

**Figure 6:**
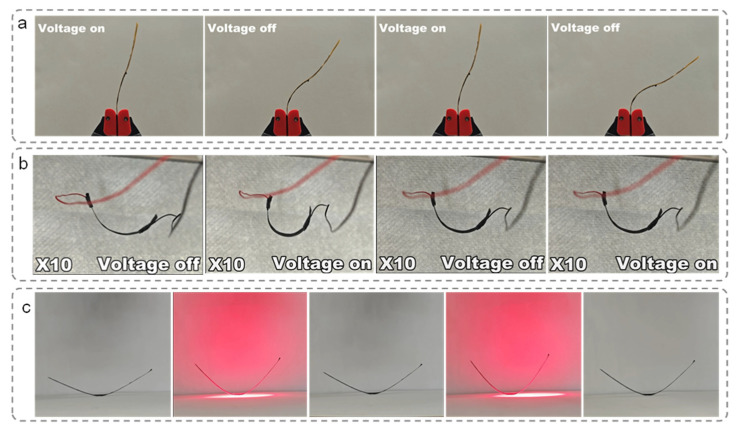
Multi-layer structure materials: (**a**) electrically driven process with one end fixed; (**b**) electrically driven process with both ends free; (**c**) light-driven process.

If the polyurethane composite material and metal material are bonded together, as shown in Figure S21a, precise driving through electrical and optical stimulation can be achieved. In this multi-layered composite structure, the metal could generate heat when electrified, thereby increasing the temperature of the polyurethane material, enabling it to exhibit shape memory behavior. During the shape-changing process, stress and energy are stored in the metal material. When the external stimulus is removed, the stored stress is released, inducing directional crystallization of the polyurethane along the stress direction, thus achieving reversible two-way shape memory behavior. Figure 6a and Figure 6b respectively demonstrate the reversible two-way shape memory process of the multi-layered structure material under one-end fixed and both-ends free conditions. In the case of electrical stimulation, the temperature of the polyurethane can be regulated by controlling the voltage, thereby achieving precise control of its deformation amplitude, as shown in Videos S4 and S5. Figure S21b shows the thermal image of the composite under electric stimulation. For optical stimulation, the m-CNTs in the polyurethane could generate heat through the photothermal effect under infrared light to increase the temperature of the polyurethane, thus enabling driving. In this process, the metal material only serves to store and release stress. Similarly, by controlling the intensity of the light, precise regulation of the polyurethane temperature can be achieved, thereby controlling its deformation amplitude, as shown in Figure 6c and Video S6.

**Figure 7:**
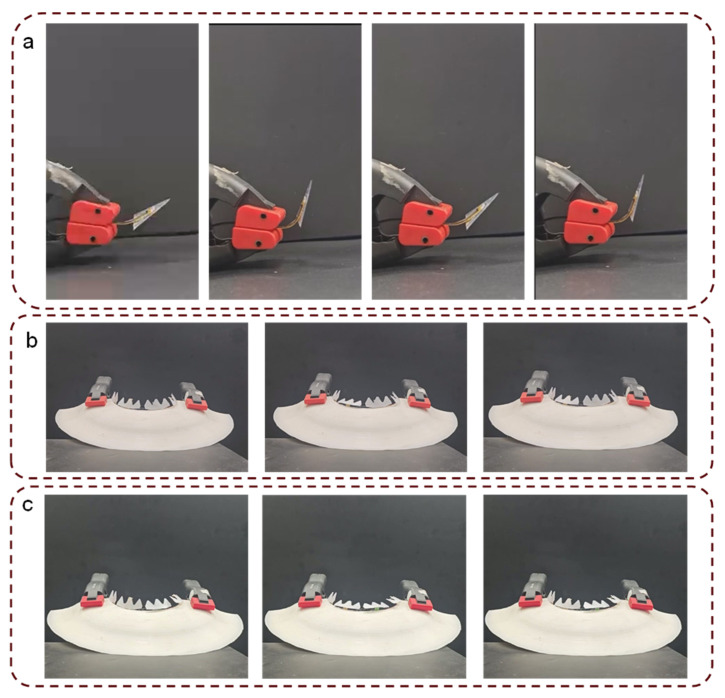
Exploration of practical applications of electrically driven multi-layer structure materials. (**a**) Reversible motion underload; (**b**) implementation of individual control in multiple drive units; and (**c**) implementation of simultaneous control in multiple driving units.

The practical application of this shape memory composite materials has been tested in different ways. Based on the excellent energy density and power density, the multi-layer structure materials could achieve reversible two-way shape memory behavior under certain loads, as shown in Figure 7a. When the composite material is combined with the load, it remains in a horizontal position at the initial state. Upon application of voltage, the material exhibits shape memory behavior and drives the load to move, bending in the vertical direction. After removing the driving voltage, the multi-layer structure material demonstrates reversible shape memory behavior, thereby driving the load back to its horizontal state. This process can be precisely controlled; the response time and response amplitude could be adjusted by magnitude the driving voltage. Based on the above control behaviors, the application scenarios of this shape memory composite material can be further expanded, such as actively controlled disturbance fluids, fiber carding devices, etc. Figure 7b,c show the multiple reversible control units prepared from multi-layer structure materials enable independent and synchronous manipulation of images. When voltage is applied only to individual control units, it can achieve independent control without affecting surrounding control units. And if voltage is applied to all control units, it can achieve synchronous control. The different control modes can meet more complex usage scenarios. Video S7 shows the entire control process behavior and drives the load to move, bending in the vertical direction. After removing the driving voltage, the multi-layer structure material demonstrates reversible shape memory behavior, thereby driving the load back to its horizontal state. This process can be precisely controlled; the response time and response amplitude could be adjusted by the magnitude of the driving voltage. Based on the above control behaviors, the application scenarios of this shape memory composite material can be further expanded, such as actively controlled disturbance fluids, fiber carding devices, etc. Figure 7b,c show the multiple reversible control units prepared from multi-layer structure materials enable independent and synchronous manipulation of images. When voltage is applied only to individual control units, it can achieve independent control without affecting surrounding control units. And if voltage is applied to all control units, it can achieve synchronous control. The different control modes can meet more complex usage scenarios. Video S7 shows the entire control process.

## 4. Conclusions

In conclusion, we report a kind of shape memory polyurethane composites, which aims to improve the mechanical properties, thermal conductivity, and shape memory performance of polyurethane by designing the material components and molecular structure. Specifically, the use of m-CNTs as a reinforcing component can significantly enhance the thermal conductivity and mechanical properties of polyurethane composites. Furthermore, this polyurethane composite exhibits excellent shape memory performance. Notably, this composite undergoes a topological transformation of its molecular chain structure at specific temperatures, enabling secondary programming of shape memory behavior and the recycling of thermosetting polyurethane materials. Additionally, due to the presence of m-CNTs, this polyurethane composite also demonstrates training enhancement, significantly improving the mechanical properties of the material after multiple cycles of stretching. Simultaneously, after the training enhancement process, the composite can enhance its shape memory characteristics, achieving stress-free two-way shape memory behavior. The composite material system also exhibits excellent cyclic stability. The excellent mechanical properties of this composite material make it suitable for use in fields such as flexible actuators and artificial muscles. In addition, its great shape memory performance can enable it to achieve reversible motion without external intervention. A dual-layered actuator was prepared using this composite material, which can achieve reversible two-way shape memory behavior under electrical and optical stimulation. By controlling the voltage and light intensity, remote and precise control of the actuator can be achieved, expanding its practical application potential and meeting the complex environmental requirements in practical applications.

## Data Availability

Data is contained within the article or Supplementary Materials.

## Supplementary Materials

The following supporting information can be downloaded at: https://www.mdpi.com/article/10.3390/polym18131582/s1. Section S1: The synthetic route of polyurethane and composite. Section S2: Calculation formulas of shape memory parameters. Figure S1: The surface of polyurethane with different filler: (a) none, (b) carbon, (c) Fe_3_O_4_ NPs and (d) m-CNTs. Figure S2: The FTIR spectrums of m-CNTs before and after modified. Figure S3: The SEM images of (a) CNTs and (b) m-CNTs. Figure S4: The SEM images of (a)–(c) PU/CBs; (d)–(f) PU/Fe_3_O_4_ and (g)–(i) PU/m-CNTs. Figure S5: TGA mechanical properties of polyurethane with different fillers. Figure S6: The surface temperature changes infrared thermography images of different polyurethane composites at 80 °C. (a) PU, (b) PU/m-CNTs, (c) PU/Carbon, (d) PU/Fe_3_O_4_. Figure S7: The surface temperature changes infrared thermography images of different polyurethane composites Under light conditions. (a) PU/m-CNTs, (b) PU/Carbon, (c) PU/Fe_3_O_4_. Figure S8: Local FTIR spectra of polyurethane composite materials with different m-CNTs contents. Figure S9: The DSC curves of shape memory polyurethane with different m-CNTs contents: (a) 0 wt%, (b) 1 wt%, (c) 3 wt%, (d) 5 wt% and (e) 7 wt%. Figure S10: The DMA curve of shape memory polymer without m-CNTs. Figure S11: Raman spectroscopy of CNTs, m-CNTs and PU/m-CNTs. Figure S12: The pictures of PU/5 wt% (a) before and (b) after stretched. Figure S13: Stress relaxation of polyurethane under 80 °C. Figure S14: (a) The DMA curve of PU/5 wt% m-CNTs; (b) Intramolecular transesterification in PU (Chain Reorganization). Figure S15: The DMA curve of PU. Figure S16: The recycling polyurethane composite. Figure S17: The (a) cyclic tensile stress-strain curves and (b) Young’s modulus of different materials. Figure S18: 500 cycles of tensile testing for PU/m-CNTs. Figure S19: DSC curve of PU/m-CNTs after cyclic stretching. Figure S20: Stress free bidirectional shape memory performance of PU/m-CNTs after 500 tensile cycles. Figure S21: (a) SEM image of the interface of multilayer structures; (b) thermal imaging pictures during the electric driving process. Table S1: The thermodynamic properties of polyurethane materials. Table S2: The shape memory properties of polyurethane materials. Table S3: The power density and energy density of polyurethane materials. Table S4: The shape memory properties of polyurethane materials after multiple cycling. Video S1: Secondary programming of shape memory process. Video S2: Quasi-bidirectional shape memory behavior. Video S3: Stress-free bidirectional shape memory behavior. Video S4: The process of electrically driven shape memory with one end fixed. The video is speeded 5 times using the Videostudio software. Video S5: The process of electrically induced shape memory with free ends. Video S6: Light-driven shape memory process. The video is speeded 10 times using the Videostudio software. Video S7: Load driven and complex control.

## Special Declaration on Ecological and Methodological Limitations

This work is only used for the study of the effect of modified carbon nanotubes on the properties of polyurethane and its shape memory performance. It currently has limitations on waste production and nanoparticle safety and cannot be used for industrial production and biological applications. Future work will place greater emphasis on the impact of materials and methods on the environment.
