# Supplementary material for: Optimized Polyurethane/CNTs Composite for Stress-Free Two-Way Shape Memory via Training Enhancement
Source: Polymers (Basel). 2026 Jun 25;18(13):1582. doi: 10.3390/polym18131582 (PMC13363853; doi:10.3390/polym18131582)
Supplement: Supplementary file 1 [file polymers-18-01582-s001.zip › Supporting Information.docx]

Supporting information

Optimized Polyurethane/CNTs Composite for Stress-Free Two-way Shape Memory via Training Enhancement

Yutong Guo,^1^ Kangkang Shi, ^2^ Yujie Chen, ^1*^ Qunfu Fan, ^1^ Dongsheng Li, ^2*^ and Hezhou Liu^1, 3^

^1^ State Key Laboratory of Metal Matrix Composites, School of Materials Science and Engineering, Shanghai Jiao Tong University, Shanghai 200240, China; xuanyin10040@sjtu.edu.cn (Y.G.); fanqunfu@sjtu.edu.cn (Q.F.); hzliu@sjtu.edu.cn (H.L.)

^2^ National Key Laboratory of Ship Vibration and Noise, China Ship Scientific Research Center, Wuxi 214082, China; shikk@cssrc.com.cn

^3^ National Engineering Research Centre of Special Equipment and Power System for Ship and Marine Engineering, Shanghai 200030, China

^*^ Correspondence: yujiechen@sjtu.edu.cn (Y.C.); lidongsheng@csscr.com.cn (D.L.)

**Section S1. The synthetic route of polyurethane and composite.**

**
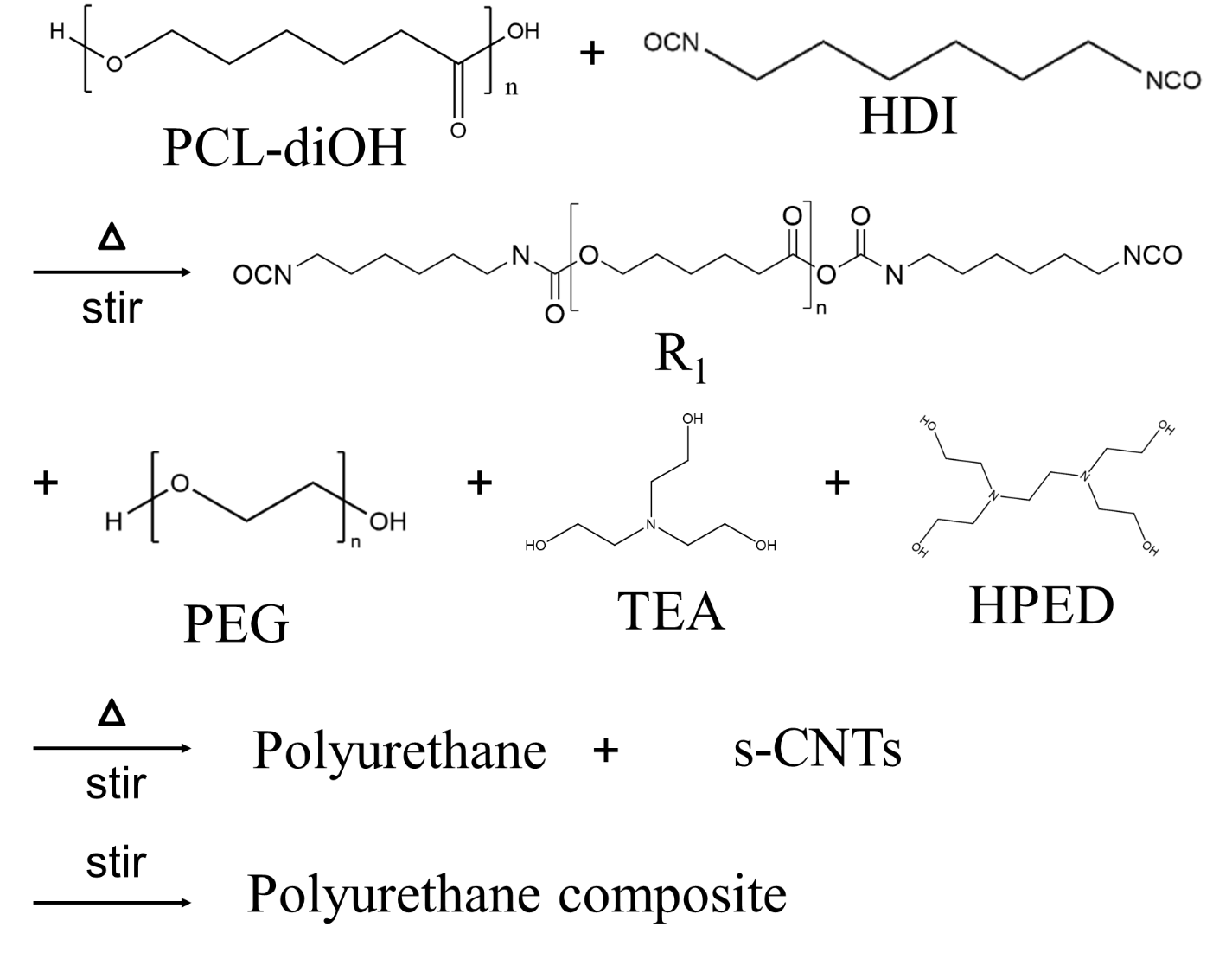
**

Firstly, polycaprolactone diol is reacted with excess hexamethylene diisocyanate (HDI) to obtain isocyanate terminated prepolymer R_1_. Then, poly(ethylene glycol) (PEG) is added to the reaction system as a chain extender, and appropriate amounts of triethanolamine (TEA) and N,N,N',N'-tetrakis(2-hydroxypropyl)ethylenediamine (HPED) are used as crosslinking agents to obtain shape memory polyurethane. Then, modified carbon nanotubes are added to the polyurethane solution in proportion to prepare high-performance polyurethane composite materials.

**Section S2. Calculation formulas of shape memory parameters.**

**Shape fixation rate**: R_f_

$R_{f}=(\frac{\varepsilon_{u(N)}}{\varepsilon_{m}})\times100\%$ $(1)$

**Shape recovery rate**: R_r_

$R_{r}=(\frac{\varepsilon_{m}-\varepsilon_{p}(N)}{\varepsilon_{m}-\varepsilon_{p}(N-1)})\times100\%$ (2)

Where ε_m_ is the maximum strain during the stretching process, ε_u_ (N) is the strain after unloading and cooling down, ε_p_ (N) is the remaining strain of the recovered shape in cycle N and ε_p_ (N-1) is the remaining strain after recovery of cycle (N-1). [1, 2]

**Energy density**：W

$W=\frac{m\times g\times\rho\times\left( L_{2}-L_{3} \right)}{M}$ (3)

Where the m represents the mass of the load, M is the mass of the polyurethane sample, and *g* is the acceleration of gravity, which value is 9.8 N kg^-1^. L_2_ is the fixation length after removing the external stress and elastic contraction, L_3_ represents the final length of the sample after the shape recovery process above the critical temperature, ρ is the density of sample.

**Power density**：P

$P=\frac{m\times g\times\left( L_{2}-L_{3} \right)}{M\times t}$ (4)

Where t is the response time of the whole shape memory behaviour under load, and other parameters are same as above formulas.

**The Arrhenius formulas:**

*κ=Aexp(−E_a_/RT​​)* (5)

where κ is the reaction rate constant, A is pre exponential factor, E_a_ is activation energy, R is Gas constant (8.314 J mol^-1^ K^-1^), and T is Absolute temperature.

we can transform it into a linear format:

*ln* $\kappa$ *=lnA-E_a_/RT* (6)

where the slope is (-E_a_/R).

**The relationship between relaxation time and κ**

$\kappa=\frac{1}{\tau}$(7)

where τ is the relaxation time. In the figure, σ is real time internal stress and σ^0^ is initial internal stress.

**Section S3：supporting videos**

**Video S1.** Secondary programming of shape memory process.

**Video S2.** Quasi-bidirectional shape memory behavior.

**Video S3.** Stress-free bidirectional shape memory behavior.

**Video S4.** The process of electrically driven shape memory with one end fixed. The video is speeded 5 times using the Videostudio software.

**Video S5.** The process of electrically induced shape memory with free ends.

**Video S6.** Light-driven shape memory process. The video is speeded 10 times using the Videostudio software.

**Video S7.** Load driven and complex control.

**Section S4：supporting figures**


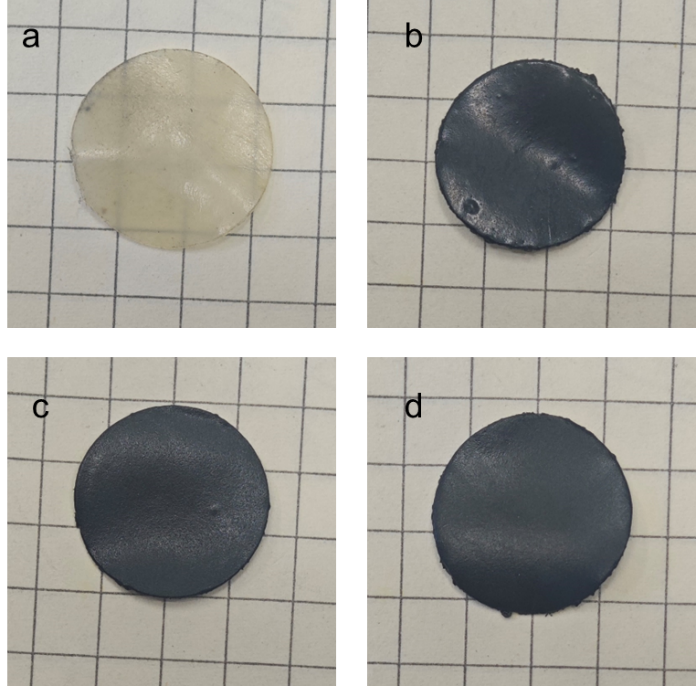


**Figure S1**. The surface of polyurethane with different filler: (a) none, (b) carbon, (c) Fe_3_O_4_ NPs and (d) m-CNTs.


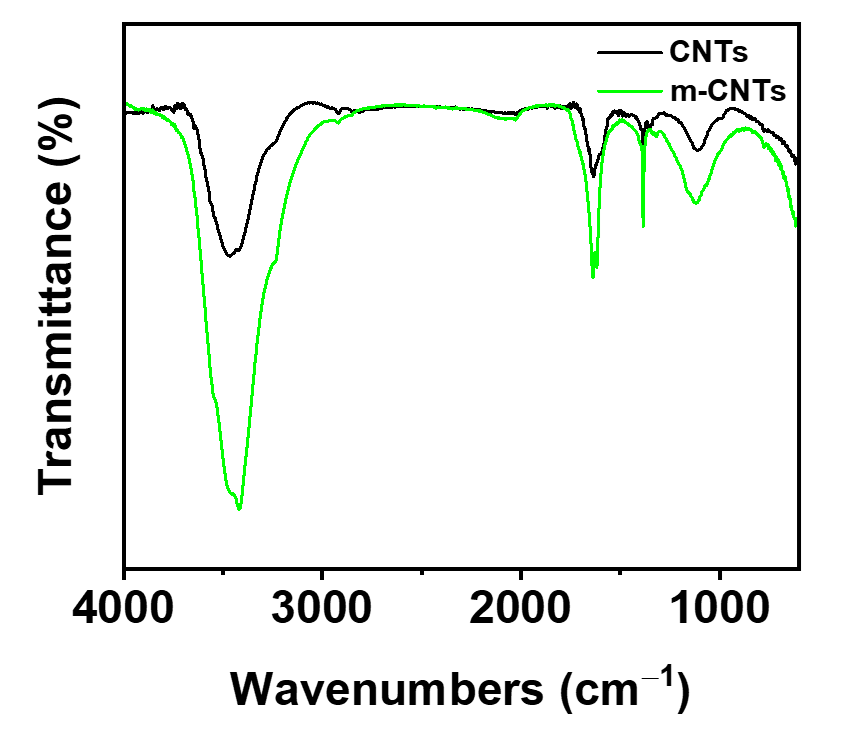


**Figure S2.** The FTIR spectrums of m-CNTs before and after modified.


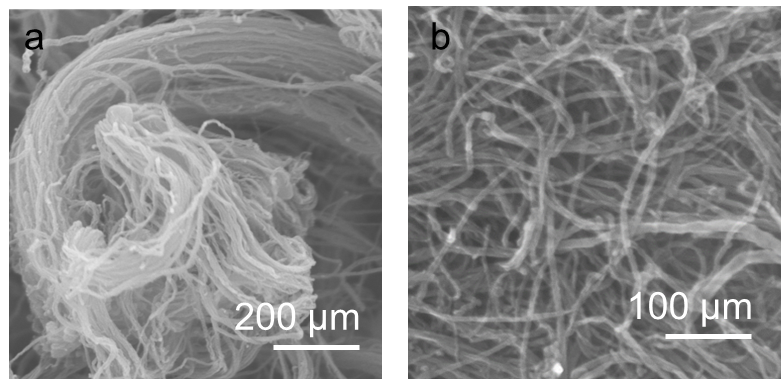


**Figure S3**. The SEM images of (a) CNTs and (b) m-CNTs.


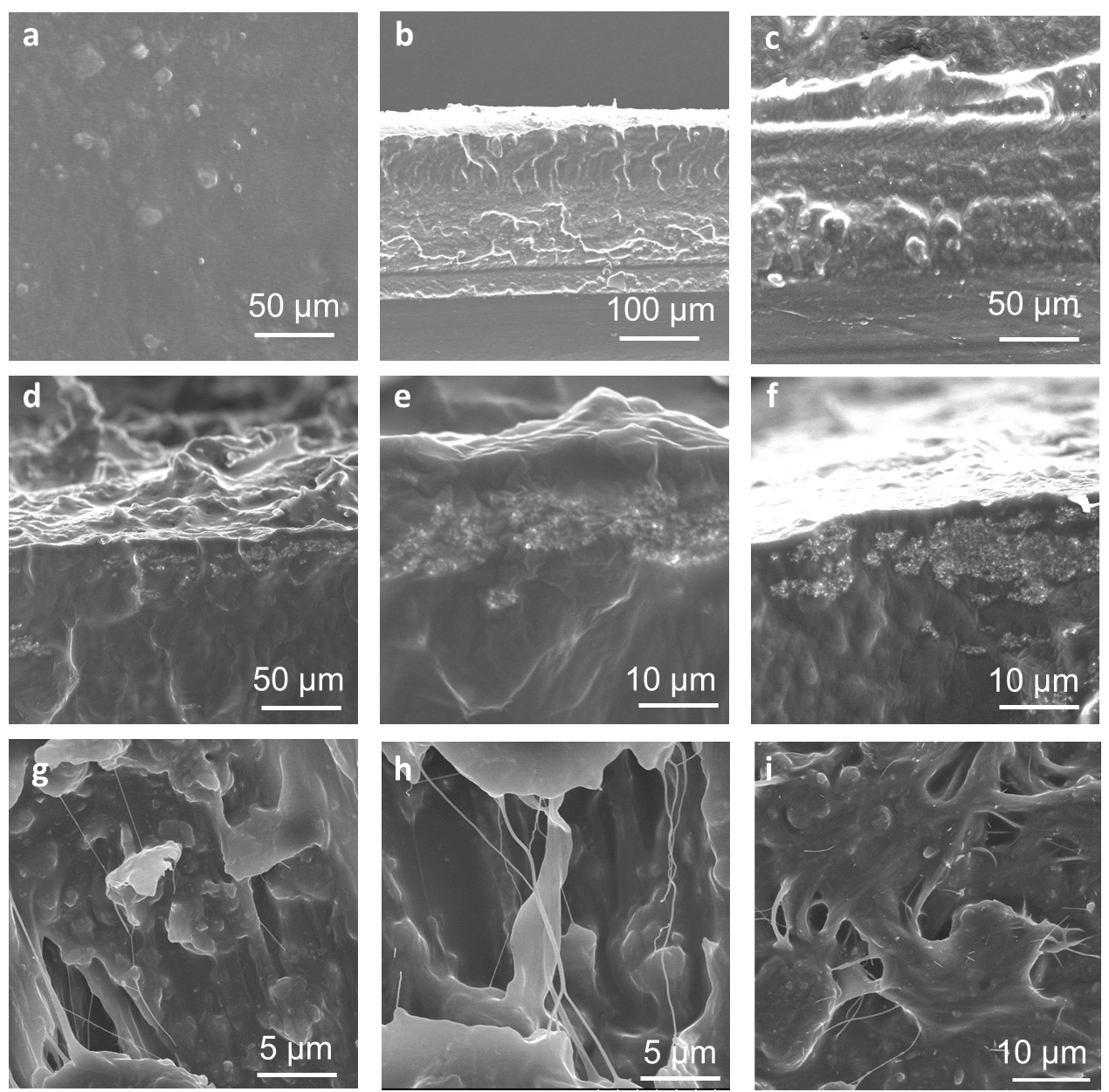


**Figure S4**. The SEM images of (a)-(c) PU/CBs; (d)-(f) PU/Fe_3_O_4_ and (g)-(i) PU/m-CNTs.


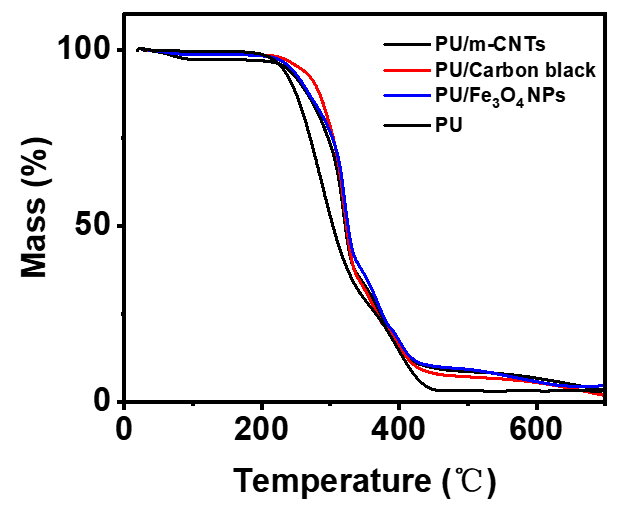


**Figure S5.** TGA mechanical properties of polyurethane with different fillers.


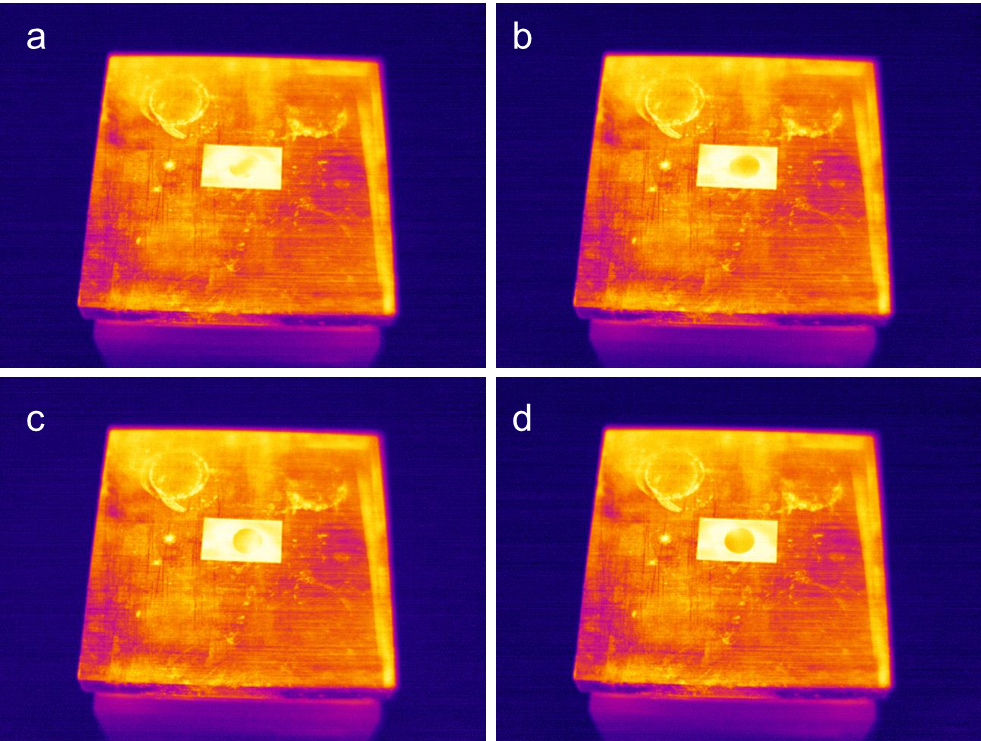


**Figure S6**. The surface temperature changes infrared thermography images of different polyurethane composites at 80 ℃. (a) PU, (b) PU/m-CNTs, (c) PU/Carbon, (d) PU/Fe_3_O_4_.


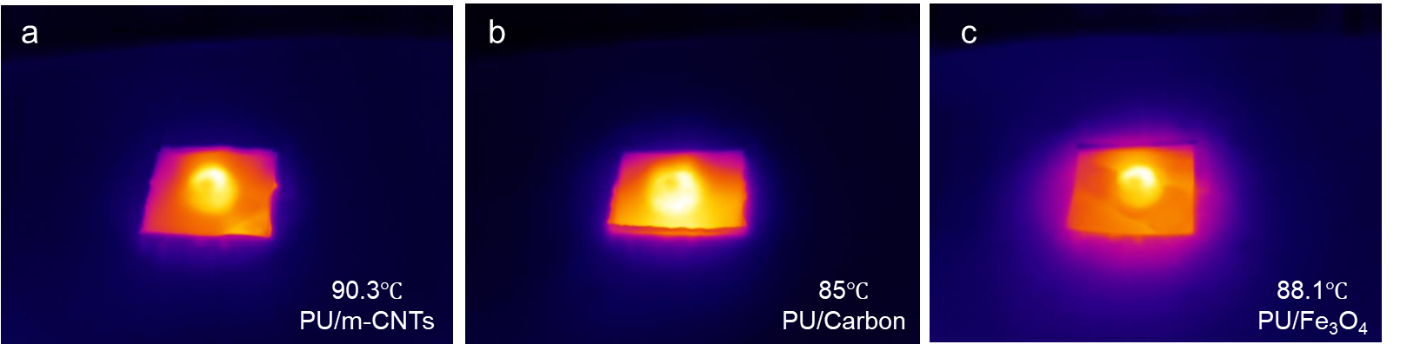


**Figure S7**. The surface temperature changes infrared thermography images of different polyurethane composites Under light conditions. (a) PU/m-CNTs, (b) PU/Carbon, (c) PU/Fe_3_O_4_.


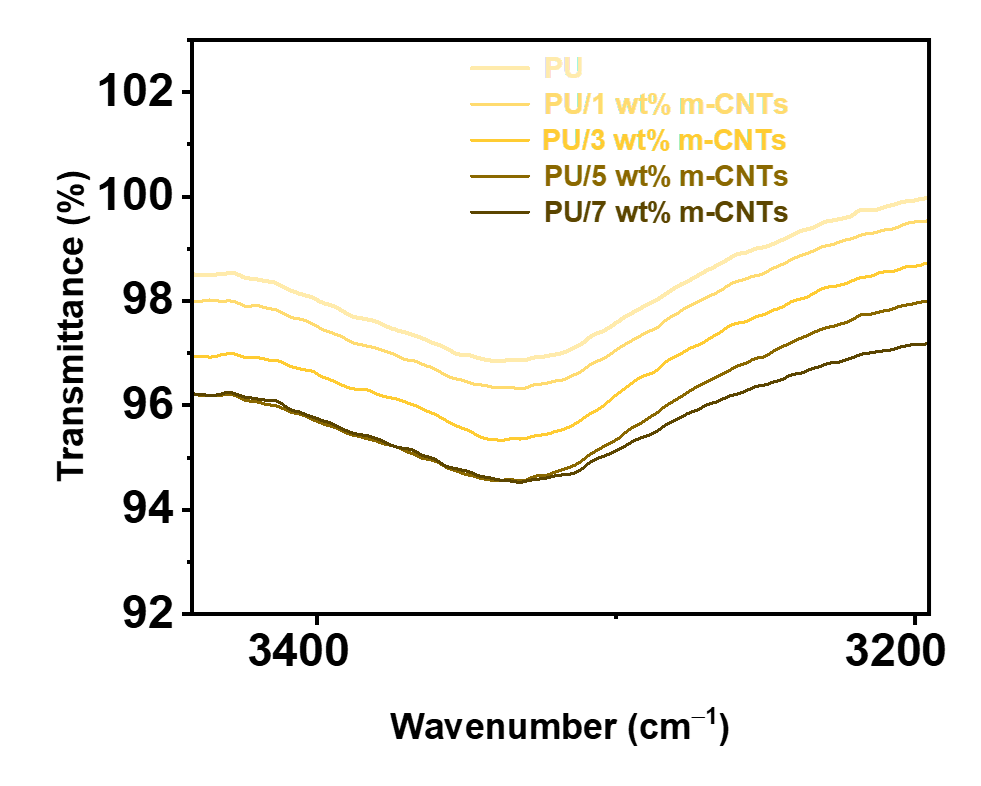


**Figure S8.** Local FTIR spectra of polyurethane composite materials with different m-CNTs contents.


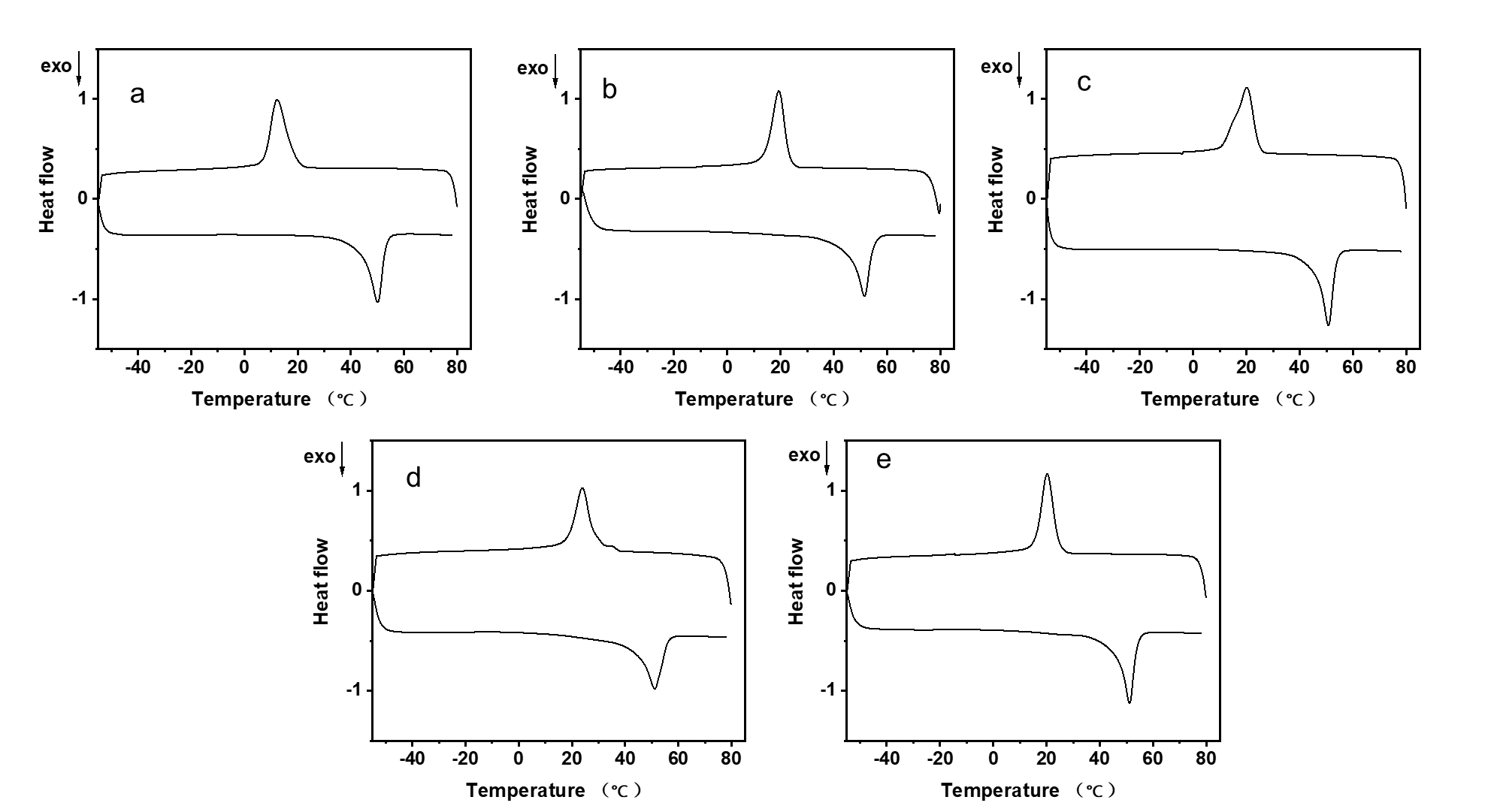


**Figure S9**. The DSC curves of shape memory polyurethane with different m-CNTs contents: (a) 0 wt%, (b) 1 wt%, (c) 3 wt%, (d) 5 wt% and (e) 7 wt%.


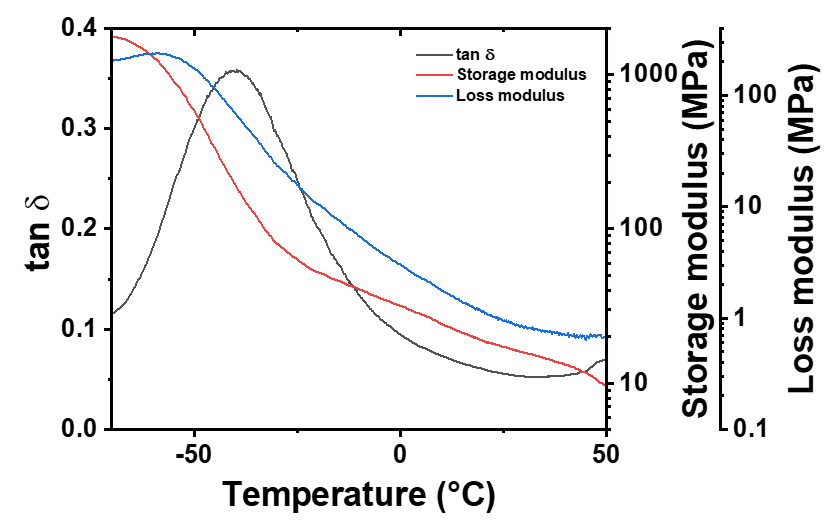


**Figure S10**. The DMA curve of shape memory polymer without m-CNTs.


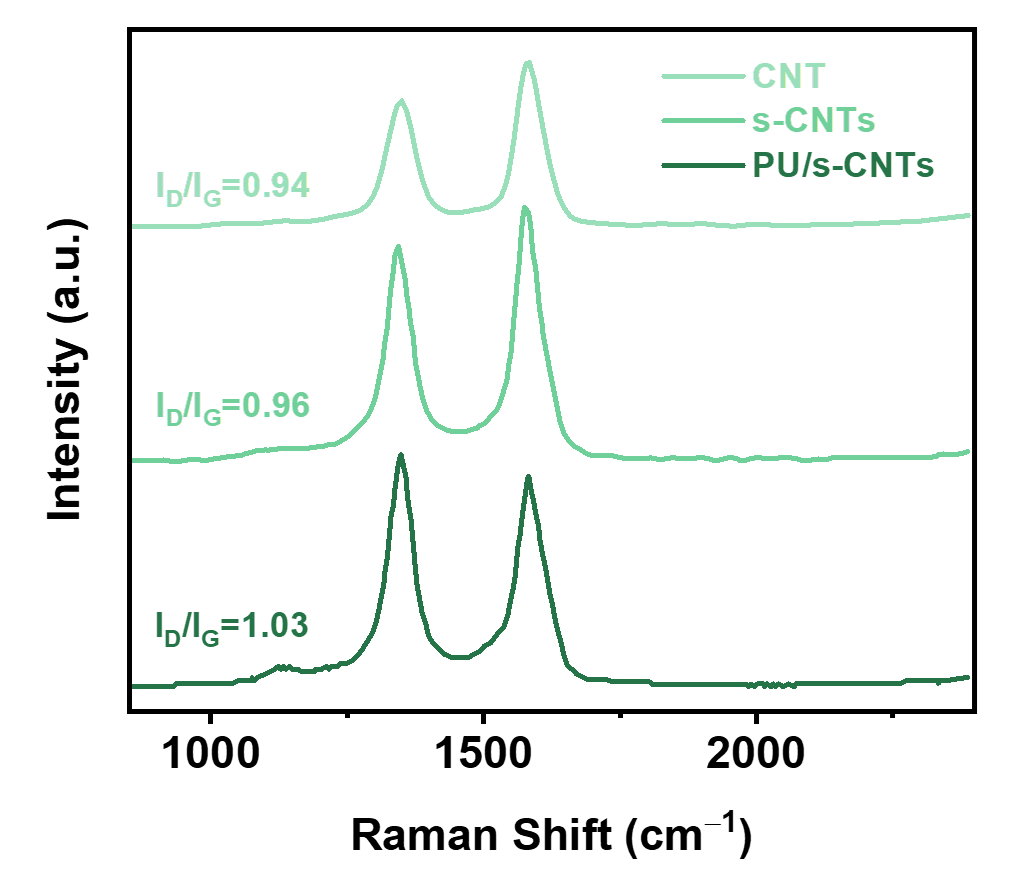


**Figure S11.** Raman spectroscopy of CNTs, m-CNTs and PU/m-CNTs.


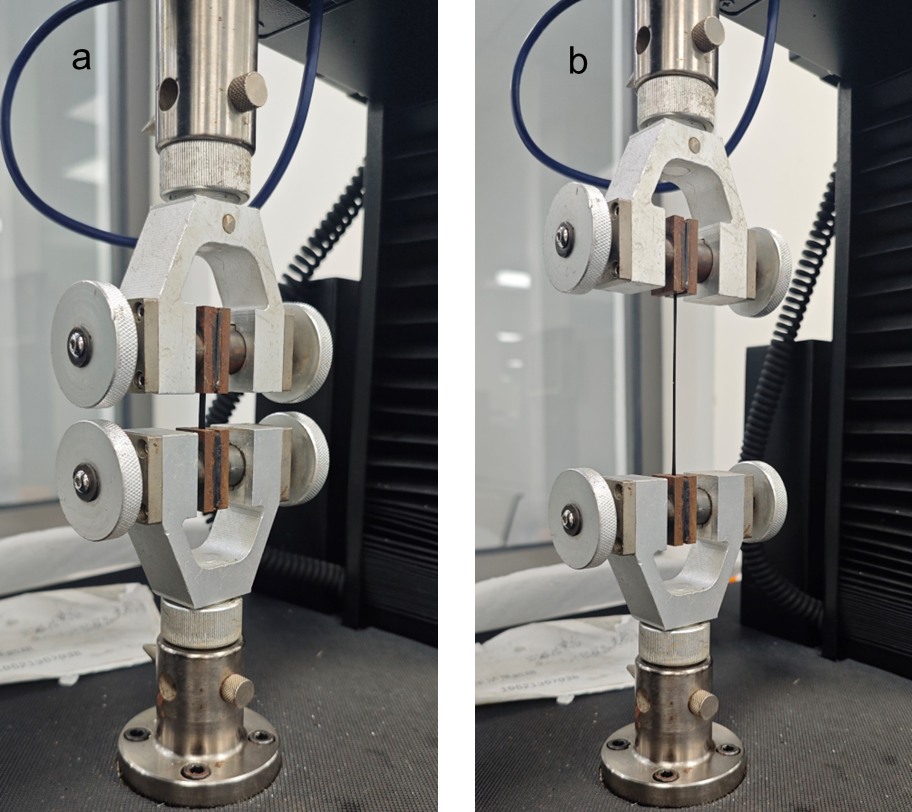


**Figure S12**. The pictures of PU/5 wt% (a) before and (b) after stretched.


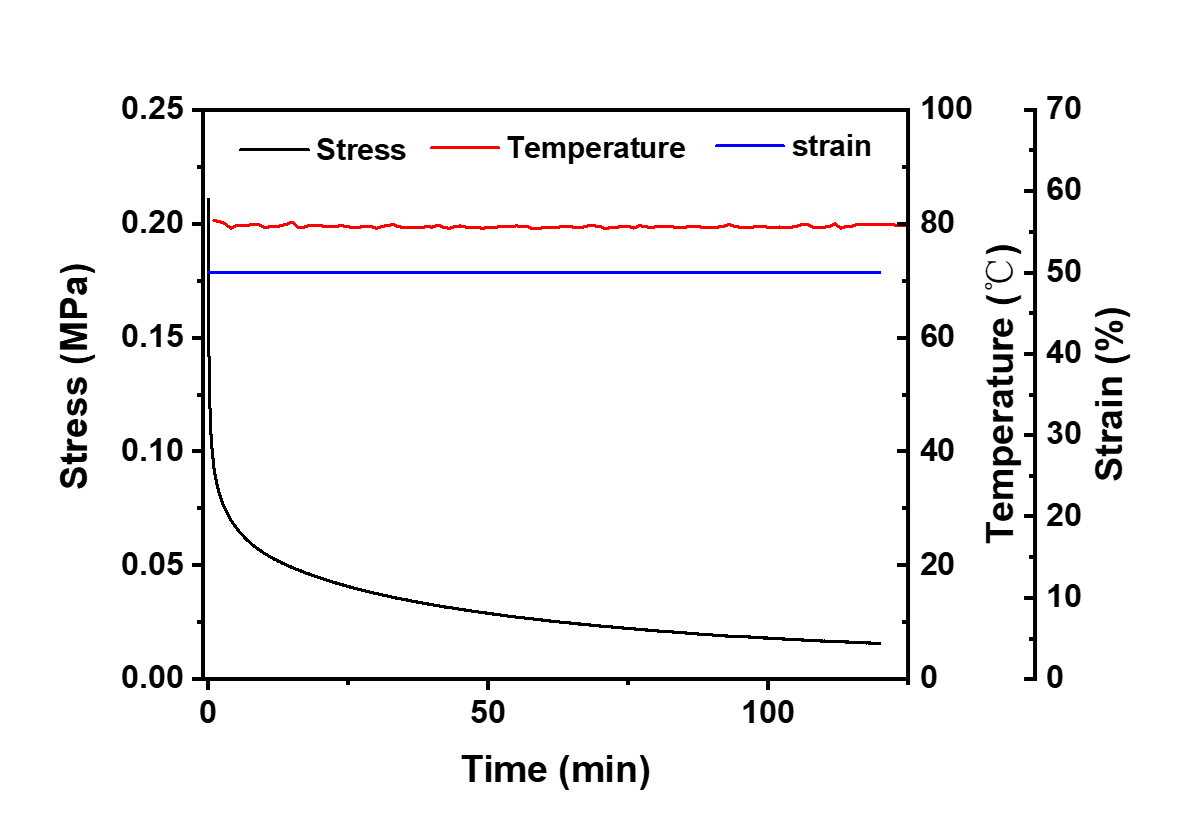


**Figure S13**. Stress relaxation of polyurethane under 80 ℃.


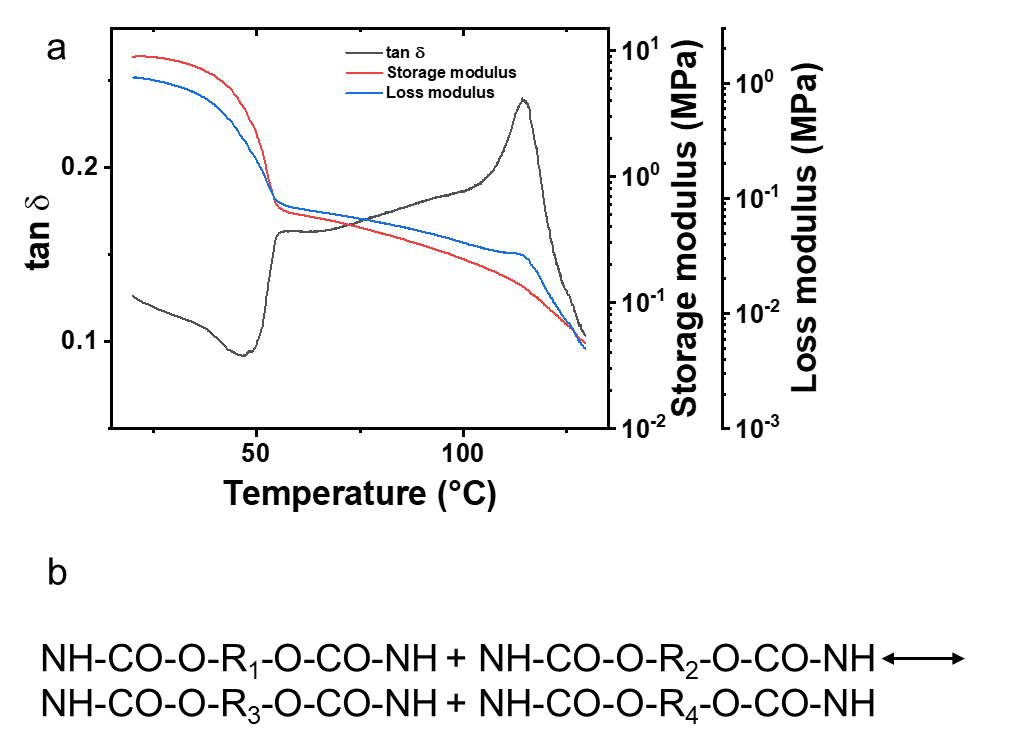


**Figure S14**. (a) The DMA curve of PU/5 wt% m-CNTs; (b) Intramolecular transesterification in PU (Chain Reorganization).


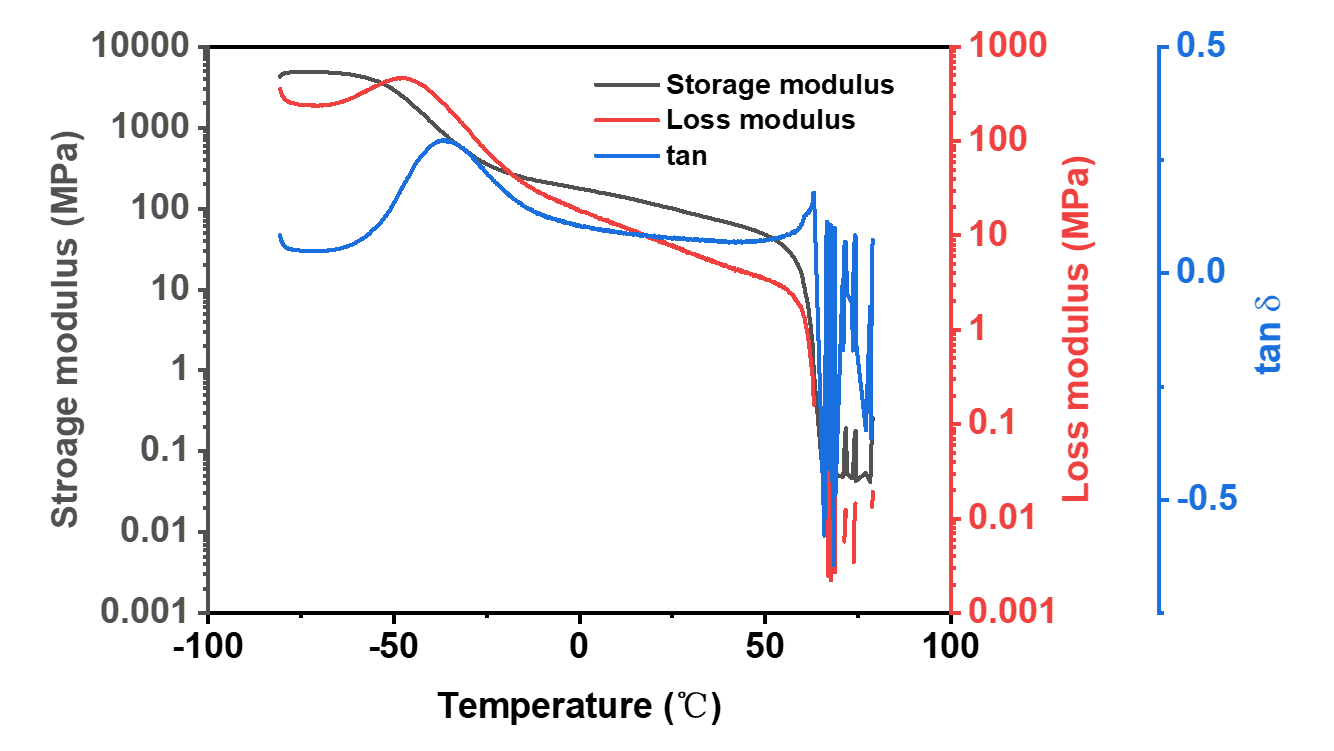


**Figure S15**. The DMA curve of PU.

**
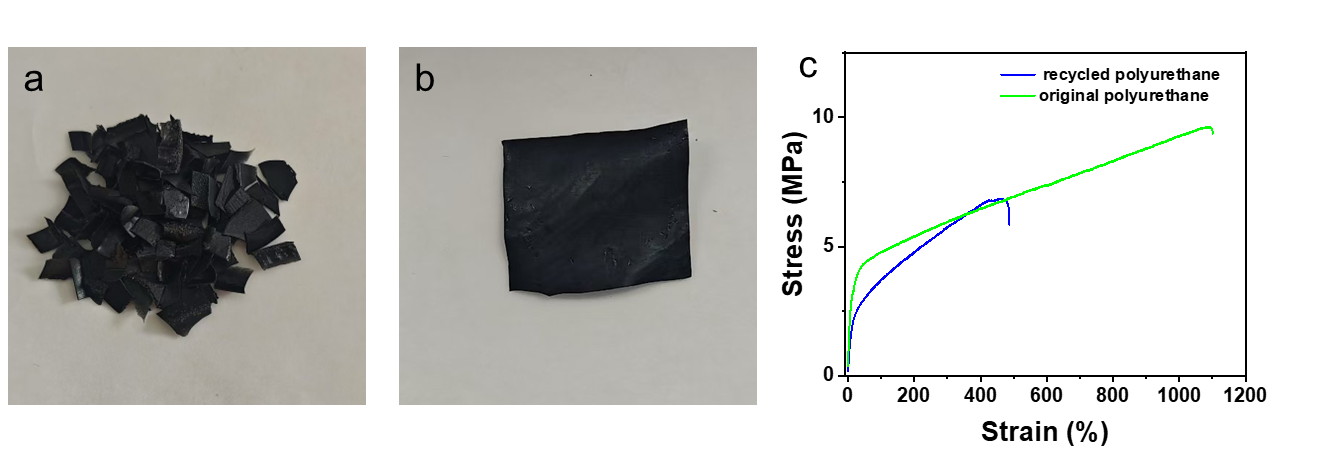
**

**Figure S16**. The recycling polyurethane composite.

**
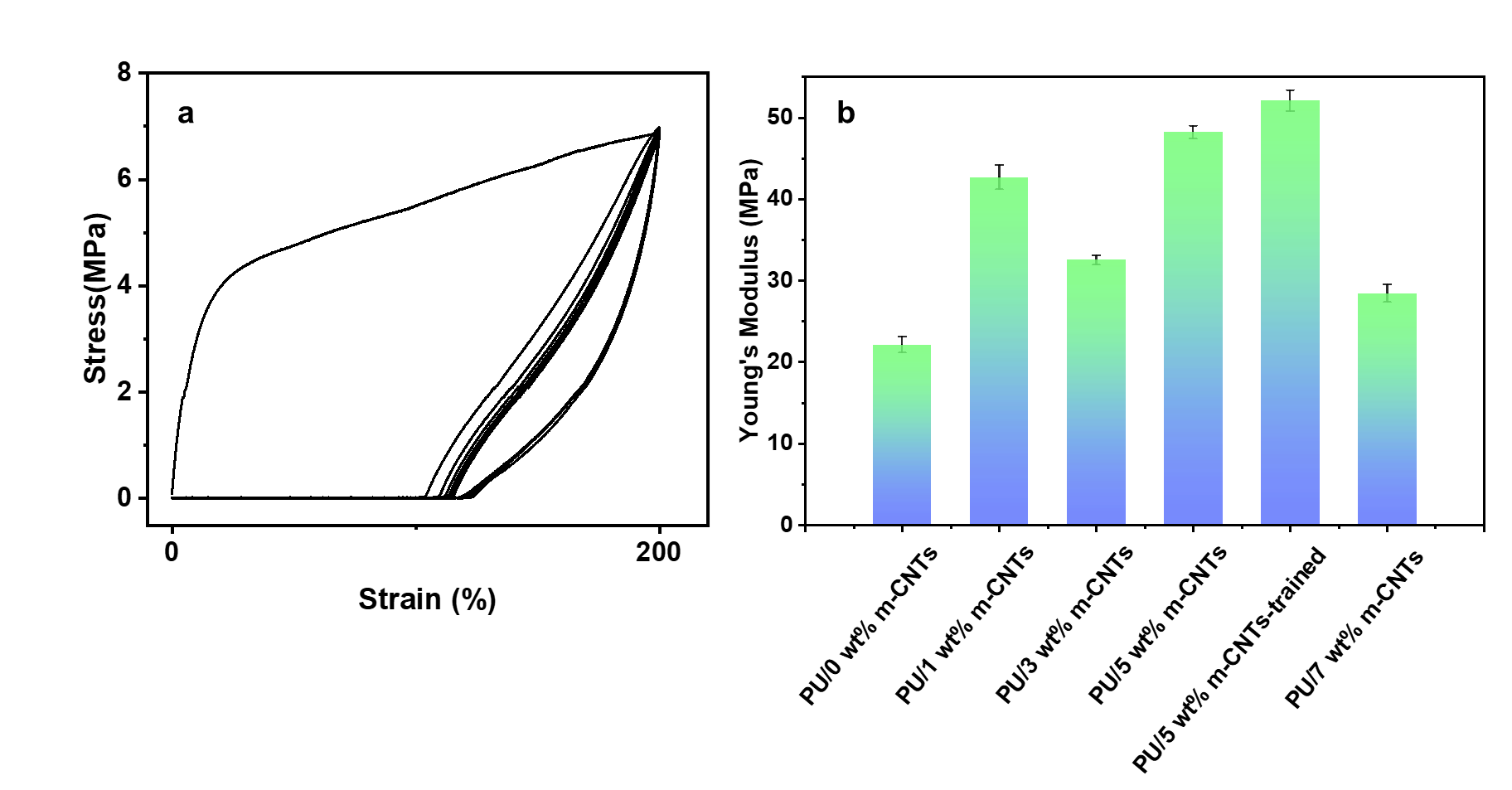
**

**Figure S17**. The (a) cyclic tensile stress-strain curves and (b) Young's modulus of different materials.


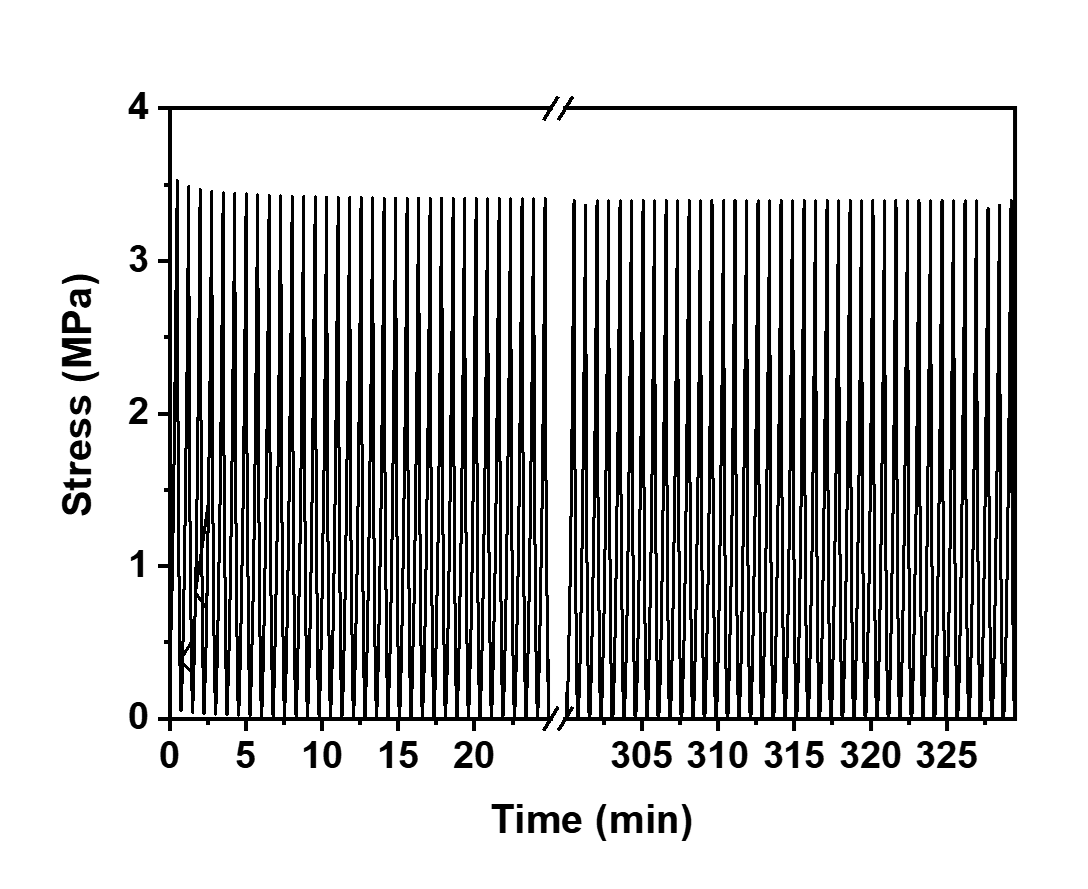


**Figure S18.** 500 cycles of tensile testing for PU/m-CNTs.


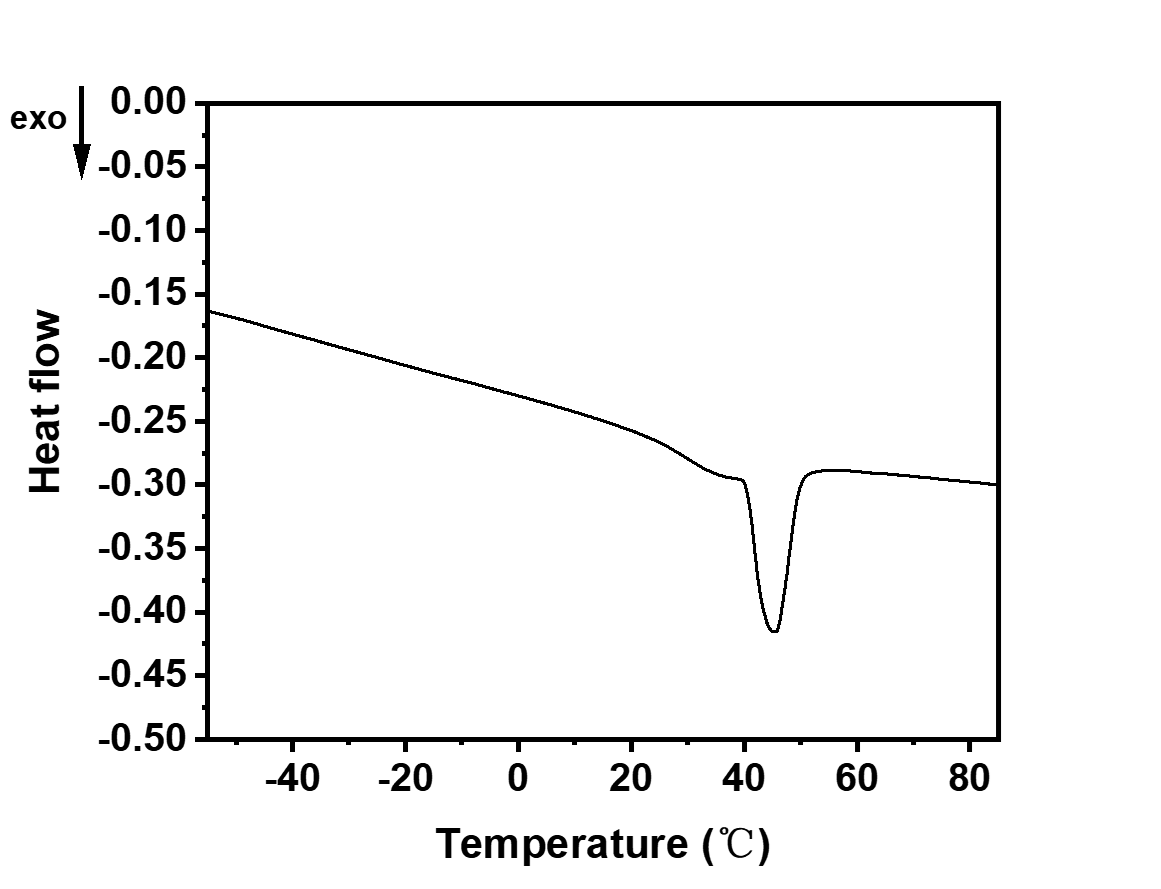


**Figure S19.** DSC curve of PU/m-CNTs after cyclic stretching


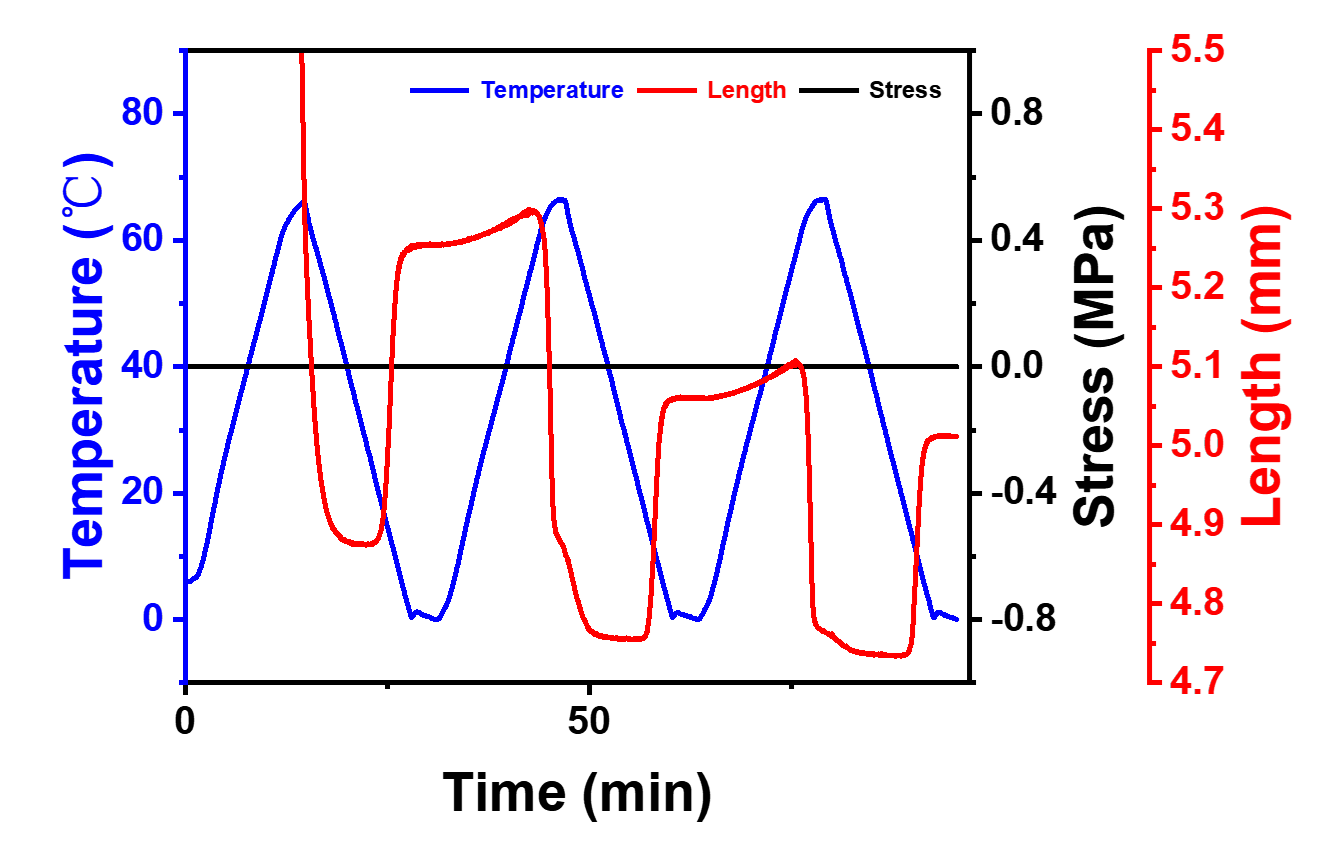


**Figure S20**. Stress free bidirectional shape memory performance of PU/m-CNTs after 500 tensile cycles.


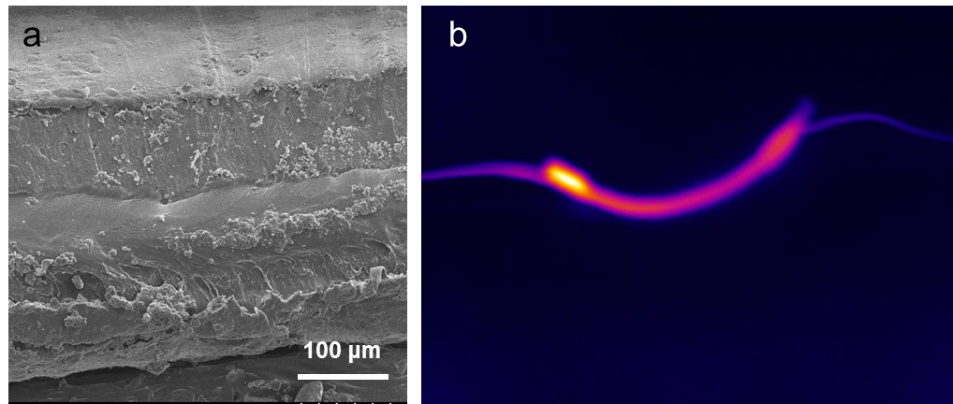


**Figure S21**. (a) SEM image of the interface of multilayer structures; (b) thermal imaging pictures during the electric driving process.

**Table S1**. The thermodynamic properties of polyurethane materials

| **Materials** | **Parameter** | **25 ℃** | **40 ℃** | **60 ℃** | **80 ℃** |
| --- | --- | --- | --- | --- | --- |
| PU/Carbon | Thermal diffusivity coefficient(mm^2^·s^-1^) | 0.143 | 0.138 | 0.128 | 0.122 |
|  | thermal conductivity（W·m^-1^K^-1^） | 0.234 | 0.240 | 0.263 | 0.220 |
|  | heat capacity C_p_（J·g^-1^K^-1^） | 1.422 | 1.515 | 1.791 | 1.568 |
| PU/Fe_3_O_4_ | Thermal diffusivity coefficient(mm^2^·s^-1^) | 0.135 | 0.133 | 0.122 | 0.117 |
|  | thermal conductivity（W·m^-1^K^-1^） | 0.255 | 0.277 | 0.295 | 0.244 |
|  | heat capacity C_p_（J·g^-1^K^-1^） | 1.639 | 1.803 | 1.980 | 1.792 |
| PU/m-CNTs | Thermal diffusivity coefficient(mm^2^·s^-1^) | 0.275 | 0.265 | 0.241 | 0.232 |
|  | thermal conductivity（W·m^-1^K^-1^） | 0.340 | 0.333 | 0.341 | 0.308 |
|  | heat capacity C_p_（J·g^-1^K^-1^） | 1.134 | 1.153 | 1.297 | 1.217 |

**Table S2**. The shape memory properties of polyurethane materials

| **Materials** | **ε_m_**  **（mm）** | **ε_u_ (N)**  **（mm）** | **ε_p_ (N)**  **（mm）** | **ε_p_ (N-1)（mm）** | **R_f_**  **（%）** | **R_r_**  **（%）** |
| --- | --- | --- | --- | --- | --- | --- |
| PU | 191.00 | 190.00 | 50.01 | 49.58 | 99.48 | 99.70 |
| PU/1 wt% m-CNTs | 94.06 | 92.15 | 34.12 | 31.11 | 97.97 | 95.22 |
| PU/3 wt% m-CNTs | 91.62 | 90.09 | 38.16 | 33.75 | 98.33 | 92.38 |
| PU/5 wt% m-CNTs | 84.35 | 83.18 | 33.79 | 31.02 | 98.61 | 94.81 |
| PU/7 wt% m-CNTs | 73.91 | 71.35 | 35.39 | 32.42 | 96.54 | 92.84 |

**Table S3**. The power density and energy density of polyurethane materials

| **Material** | **L_2_**  **（mm）** | **L_3_**  **（mm）** | **m**  **（g）** | **M**  **（g）** | **t**  **（s）** | **ρ**  **（g·cm^-3^）** | **W**  **(J·kg^-1^）** | **P**  **(W·kg^-1^）** |
| --- | --- | --- | --- | --- | --- | --- | --- | --- |
| PU | 92.63 | 49.24 | 0.102 | 4.87 | 1 | 1.017 | 336.63 | 317.57 |
| PU /1 wt% m-CNTs | 105.74 | 32.17 | 0.068 | 4.87 | 1 | 1.026 | 220.89 | 197.22 |
| PU /3 wt% m-CNTs | 117.06 | 46.59 | 0.057 | 4.87 | 1 | 1.065 | 496.69 | 443.47 |
| PU /5 wt% m-CNTs | 108.20 | 31.15 | 0.053 | 4.87 | 1 | 1.091 | 679.39 | 468.55 |
| PU /7 wt% m-CNTs | 93.40 | 30.79 | 0.049 | 4.87 | 1 | 1.095 | 468.89 | 308.48 |

**Table S4**. The shape memory properties of polyurethane materials after multiple cycling

| **Materials** | **ε_m_**  **（mm）** | **ε_u_ (N)**  **（mm）** | **ε_p_ (N)**  **（mm）** | **ε_p_ (N-1)（mm）** | **R_f_**  **（%）** | **R_r_**  **（%）** |
| --- | --- | --- | --- | --- | --- | --- |
| PU | 100 | 96.37 | 32.16 | 33.21 | 96.37 | 98.45 |
| PU/1 wt% m-CNTs | 100 | 94.74 | 33.23 | 36.75 | 94.74 | 94.73 |
| PU/3 wt% m-CNTs | 100 | 93.81 | 34.16 | 36.48 | 93.81 | 96.47 |
| PU/5 wt% m-CNTs | 100 | 93.97 | 36.45 | 39.62 | 93.97 | 95.01 |
| PU/7 wt% m-CNTs | 100 | 86.37 | 37.17 | 40.73 | 86.37 | 94.33 |
